# A SWOT (Strengths, Weaknesses, Opportunities, and Threats) Analysis of ChatGPT in the Medical Literature: Concise Review

**DOI:** 10.2196/49368

**Published:** 2023-11-16

**Authors:** Daniel Gödde, Sophia Nöhl, Carina Wolf, Yannick Rupert, Lukas Rimkus, Jan Ehlers, Frank Breuckmann, Timur Sellmann

**Affiliations:** 1 Department of Pathology and Molecularpathology Helios University Hospital Wuppertal Witten/Herdecke University Witten Germany; 2 Faculty of Health Witten/Herdecke University Witten Germany; 3 Department of Didactics and Education Research in the Health Sector Faculty of Health Witten/Herdecke University Witten Germany; 4 Department of Cardiology and Vascular Medicine West German Heart and Vascular Center Essen University Duisburg-Essen Essen Germany; 5 Department of Cardiology, Pneumology, Neurology and Intensive Care Medicine Klinik Kitzinger Land Kitzingen Germany; 6 Department of Anaesthesiology I Witten/Herdecke University Witten Germany; 7 Department of Anaesthesiology and Intensive Care Medicine Evangelisches Krankenhaus BETHESDA zu Duisburg Duisburg Germany

**Keywords:** ChatGPT, chatbot, artificial intelligence, education technology, medical education, machine learning, chatbots, concise review, review methods, review methodology, SWOT

## Abstract

**Background:**

ChatGPT is a 175-billion-parameter natural language processing model that is already involved in scientific content and publications. Its influence ranges from providing quick access to information on medical topics, assisting in generating medical and scientific articles and papers, performing medical data analyses, and even interpreting complex data sets.

**Objective:**

The future role of ChatGPT remains uncertain and a matter of debate already shortly after its release. This review aimed to analyze the role of ChatGPT in the medical literature during the first 3 months after its release.

**Methods:**

We performed a concise review of literature published in PubMed from December 1, 2022, to March 31, 2023. To find all publications related to ChatGPT or considering ChatGPT, the search term was kept simple (“ChatGPT” in AllFields). All publications available as full text in German or English were included. All accessible publications were evaluated according to specifications by the author team (eg, impact factor, publication modus, article type, publication speed, and type of ChatGPT integration or content). The conclusions of the articles were used for later SWOT (strengths, weaknesses, opportunities, and threats) analysis. All data were analyzed on a descriptive basis.

**Results:**

Of 178 studies in total, 160 met the inclusion criteria and were evaluated. The average impact factor was 4.423 (range 0-96.216), and the average publication speed was 16 (range 0-83) days. Among the articles, there were 77 editorials (48,1%), 43 essays (26.9%), 21 studies (13.1%), 6 reviews (3.8%), 6 case reports (3.8%), 6 news (3.8%), and 1 meta-analysis (0.6%). Of those, 54.4% (n=87) were published as open access, with 5% (n=8) provided on preprint servers. Over 400 quotes with information on strengths, weaknesses, opportunities, and threats were detected. By far, most (n=142, 34.8%) were related to weaknesses. ChatGPT excels in its ability to express ideas clearly and formulate general contexts comprehensibly. It performs so well that even experts in the field have difficulty identifying abstracts generated by ChatGPT. However, the time-limited scope and the need for corrections by experts were mentioned as weaknesses and threats of ChatGPT. Opportunities include assistance in formulating medical issues for nonnative English speakers, as well as the possibility of timely participation in the development of such artificial intelligence tools since it is in its early stages and can therefore still be influenced.

**Conclusions:**

Artificial intelligence tools such as ChatGPT are already part of the medical publishing landscape. Despite their apparent opportunities, policies and guidelines must be implemented to ensure benefits in education, clinical practice, and research and protect against threats such as scientific misconduct, plagiarism, and inaccuracy.

## Introduction

ChatGPT is a 175-billion-parameter natural language processing model that can generate conversation-style responses to user input. Parameters are the numerical values that determine how a neural network processes input data and produces output data. They are learned from data during the training process, and they encode the knowledge and skills of the model [1,2]. Since its launch by OpenAI, ChatGPT has stimulated widespread conversation and momentum across various human sciences, including medicine, as demonstrated by over 1250 publications related to ChatGPT indexed in PubMed in 2023 so far. General interest is reflected by the rapid increase in user numbers within a very short time—after just 1 week, ChatGPT had reached the 1 million-user mark, and by January 2023, the number of users had exceeded 100 million [1,3]. It enables humans to interact and discuss with an artificial intelligence (AI) chatbot on a broad range of topics. Since it became known that ChatGPT was able to pass the United States Medical Licensing Examination, performing at a level comparable to a third-year medical student [4], it has garnered even more interest. Preliminary evidence shows that ChatGPT has promising applications across the clinical workflow, especially regarding clinical decision-making, narrowing the final diagnosis, and clinical management [5]. Moreover, ChatGPT and similar generative AI tools have specific applications within medical education, including clinical vignette generation and communication training with AI virtual patients [5-7]; however, it poses several challenges that universities need to carefully address [8]. Tested on the verbal scale of the Wechsler Adult Intelligence Scale (WAIS), an intelligence quotient of 155 was demonstrated for ChatGPT [9]. When the authors asked ChatGPT to explain its role in medical literature,” it responded as follows:

As an AI language model, ChatGPT’s role in medical literature is to provide information and insights related to health care and medicine based on its knowledge of the subject matter. ChatGPT can help researchers and health care professionals by providing quick access to information on a wide range of medical topics, including symptoms, diagnoses, treatments, and outcomes. ChatGPT can also assist in generating summaries and abstracts of medical articles and papers, making it easier for health care professionals to keep up with the latest developments in their field. Additionally, ChatGPT can assist in developing natural language processing applications that can improve the efficiency and accuracy of medical data analysis and interpretation. However, it’s important to note that ChatGPT is not a substitute for medical professionals and should not be used to make medical decisions. It is always recommended to consult with a licensed health care provider for medical advice and treatment.

ChatGPT response

Although these features are promising, the technology is still vulnerable to misuse and abuse due to the lack of regulation. It can be tempting to save time by creating manuscripts using ChatGPT. However, there is a risk that false information will be published or used, which may compromise patient safety. Since its release, over 200 papers have addressed issues using the search term “ChatGPT.” This review aimed to analyze the role of ChatGPT in the medical literature during the first 3 months after its release. The main focus is on how ChatGPT can help authors with research, data analysis, and even writing medical papers, although articles, publishing journals, and authors were studied in more detail. To provide an overview, uncover more information about its potential, and create hypotheses for further research, we undertook this concise review on the role of ChatGPT including a SWOT (strengths, weaknesses, opportunities, and threats) analysis to define its potential, especially for medical publishing.

## Methods

### Study Design: Search Strategy and Selection Criteria

To extract all publications related to or considering ChatGPT from December 1, 2022, to March 31, 2023, we searched PubMed for the Medical Subject Headings (MeSH) term “ChatGPT” in AllFields on April 1, 2023. All full-text publications in German or English published during this period were included. In the next step, we excluded articles that were not written about the use of ChatGPT but where ChatGPT acted as coauthor or where ChatGPT was used to support the text generation (Textbox 1). The final reference list was generated based on relevance to the broad scope of this review.

**Inclusion and exclusion criteria for the literature search.**

**Inclusion criteria**

- Fully retrievable articles
- Published in English and German
- Published between December 1, 2022, and March 31, 2023
- Human author(s)

**Exclusion criteria**

- Nonretrievable articles
- Published in languages other than English or German
- Published before December 1, 2022, and after March 31, 2023
- Authored by ChatGPT

### Data Analysis

All accessible publications were evaluated according to the following specifications by the authors: title, publication date, PubMed ID, author (including publication experience, specialization), journal title (including specialization), impact factor, publication modus, article type, study type, publication speed, type of ChatGPT integration, content analysis (evidence for strengths, weaknesses, opportunities, and/or threats), and further comments. For evaluation, the full texts were divided between the authors. Each publication was first evaluated and categorized by an author concerning the given criteria. The results were documented in a collaborative table. To increase reliability and validity, the results were then reviewed by a second author. In case of differences in the evaluation, a discussion between the 2 authors took place until a consensus was reached. If it could not be reached, the evaluation was performed by a third author, and a majority decision was made.

### Articles

Publications were primarily classified according to the specifications of PubMed. For better comprehensibility, a “studies” category was created, defined as “a method of research in which a problem is identified, relevant data are gathered, a hypothesis is formulated, and the hypothesis is empirically tested.” This category allowed for differentiation from nonempirical publications such as editorials. All identified articles were scanned for qualitative (ie, a collection of text-based data, such as interviews and focus groups, usually hypothesis-generating) versus quantitative (ie, a collection of number-based data, such as measurements, questionnaires with associated statistics, usually hypothesis-testing) content. We also chose to discriminate between mixed methods research (ie, a combination of qualitative and quantitative content) and reviews and meta-analysis. Article contents were analyzed in reporting on the use or actual, partial, or full use of ChatGPT in the drafting of the article. In this context, attention was also paid to the correlation between the share of ChatGPT in the preparation of the manuscript (not at all, partially, completely) and the achievable impact factor. The conclusions of the articles were entered in the table in the short form to allow for later SWOT analysis. The publishing speed and the presence of preprint were documented. To better compare the course of the number of actual published papers on ChatGPT, an article count was displayed by week and compared to weekly article releases during the COVID-19 outbreak, an impactful event in current medical history.

### Journals

Journals publishing articles on ChatGPT were evaluated regarding title, discipline of natural science, actual impact factor, and open access versus traditional publishing. The publishing speed (including preprint servers) was only included in the analysis if given in the paper itself or alongside the date of submission and publication.

### Authors

To obtain further information about the authors publishing on the topic, the number of first and last authorships of each first author in PubMed was determined for the years between 2020 and 2022. In addition, the specialty of each first author was also determined via the affiliations in the paper, PubMed, or via the ORCID (Open Researcher and Contributor ID).

### SWOT Analysis

The authors broke into groups of two to work on the first part of the SWOT analysis. They examined all the documented comments to determine if they fit into their area. A consensus had to be reached between the authors. If this was not possible, it was noted. Then, the domains were swapped (strengths versus threats and weaknesses versus opportunities), and the previous divisions were reviewed. Particular attention was paid to comments that did not previously reach consensus. Each comment was thus assigned by 4 authors. If consensus decisions could not be reached, the majority decision counted.

### Statistical Analysis

The primary endpoint was strengths, weaknesses, opportunities, and threats of ChatGPT use in medical literature, with subcategories of author, article, and journal type. All data were analyzed on a descriptive basis. Data were presented as means and SD unless otherwise stated. Statistical analysis was performed descriptively using Microsoft Excel for Office 365 (Microsoft Corp) and PSPP (GNU Project, Free Software Foundation). The Student *t* test, Levene test, and Mann-Whitney *U* test were applied as appropriate. *P*<.05 was considered to represent statistical significance.

## Results

### Overview

**Figure 1:**
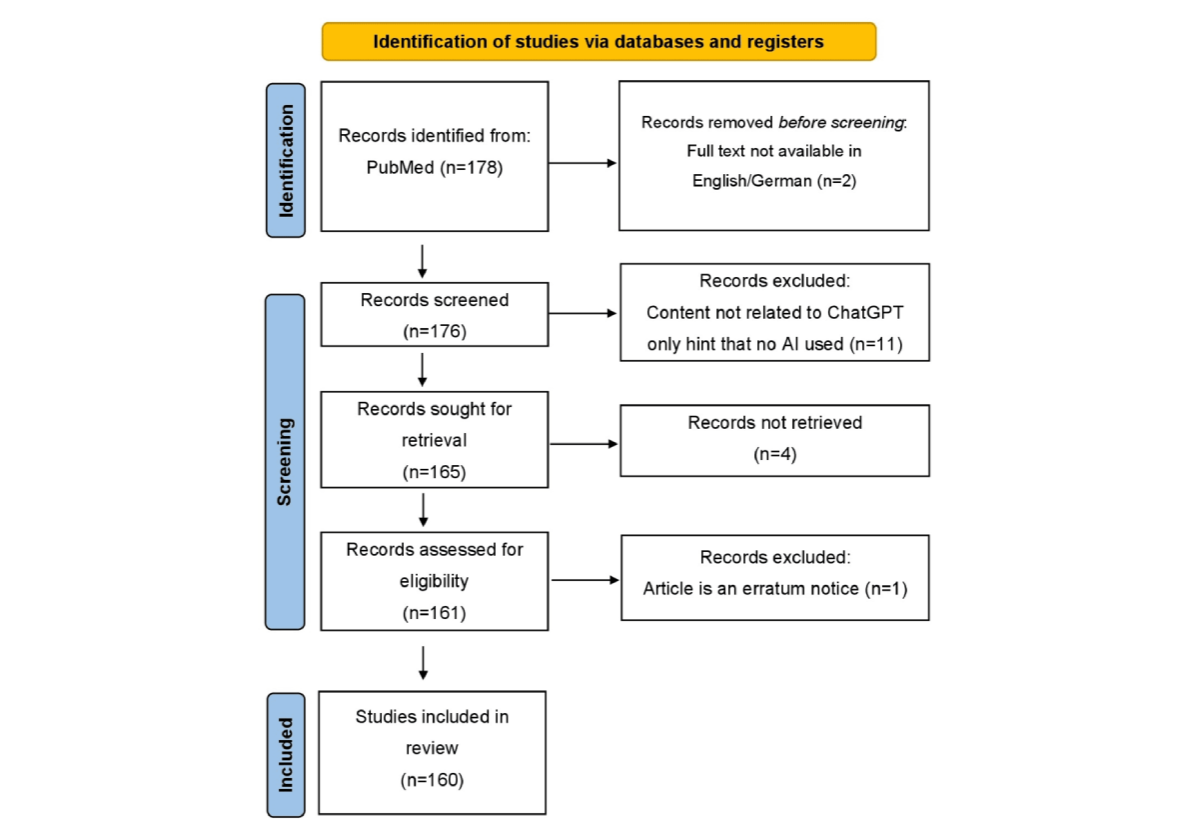
PRISMA (Preferred Reporting Items for Systematic Reviews and Meta-Analyses) flowchart of studies related to ChatGPT.

From December 1, 2022, to March 31, 2023, a total of 178 papers using the search term “ChatGPT” were published in PubMed; among them, 6 (3.4%) were published in December 2022, 16 (9%) in January 2023, 68 (38.2%) in February 2023, and 88 (49.4%) in March 2023. After a thorough human review, 18 papers had to be excluded, 11 because they were written with but not about ChatGPT, 4 that were not retrievable as full text, and 2 that were neither written in English nor German. One paper was just an erratum note. Figure 1 shows the PRISMA (Preferred Reporting Items for Systematic Reviews and Meta-Analyses) flowchart of ChatGPT-related publications (modified after [10]).

### Articles

The majority of the articles were brief statements like editorials, or letters to the editor (n=77, 48.1%). Essays or commentaries (n=43, 26.9%) represented the second largest portion of the articles. Research studies not further specifiable (n=21, 13.3%), reviews, news, case reports (each n=6, 3.8%), and meta-analyses (n=1, 0.6%) were less frequent. No randomized controlled trial (RCT) could be identified. Table 1 shows the distribution of article types according to the specifications of PubMed publication type. Of all the articles, 80% (n=128) contained nonempirical data and 20% (n=32) contained empirical data. Among these, 6.9% (n=11) were qualitative, 8.8% (n=14) were quantitative, and 1.9% (n=3) were mixed methods. Regarding the proportion of ChatGPT within the article, 11.9% (n=19) of all articles were written at least partially with ChatGPT. The average impact factors are displayed in Table 2.

**Table 1:** Distribution of article types according to PubMed publication type (N=160)^a^.

Article type	Values, n (%)
Editorial	77 (48.1)
Opinion/essay	43 (26.9)
Study	21 (13.1)
Case report	6 (3.8)
News	6 (3.8)
Review	6 (3.8)
Meta-analysis	1 (0.6)

^a^The name of the publication was adopted analogously to the terminology in PubMed. “News” in this context means information or reports about recent events, whereas “study” means an organized experiment.

**Table 2:** Average impact of articles written with or without ChatGPT.

Articles with an impact factor	Values, n (%)	Median (IQR)	Range
Written without ChatGPT	120	5.622^a^ (3.282-13.89)	0.646-96.216
Written with ChatGPT	18	4.403^a^ (3.499-7.188)	1.15-29.983

^a^Indicates nonsignificant *P* values. The Levene test *(P*=.001) indicated that the *t* test, although primarily significant (*P*=.003), was not robust, so statistical significance was also calculated using the Mann-Whitney *U* test (*P*=.4).

**Figure 2:**
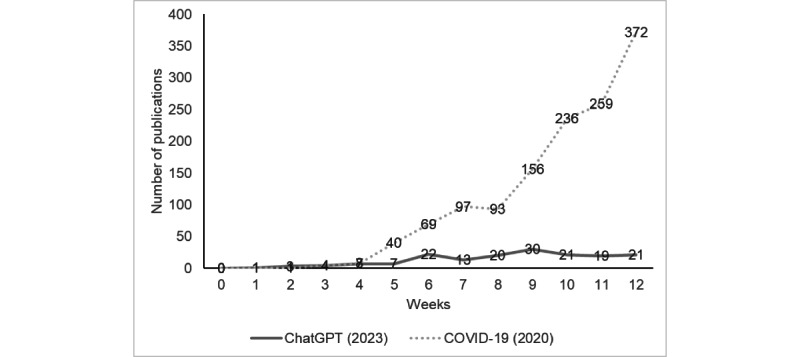
Weekly publications on ChatGPT compared to COVID-19. Data for COVID-19–related publications were taken from Kambhampati et al [11]. During the first 4 weeks, there was no marked differences in publications (ChatGPT/COVID-19, week 1: 1/1, week 2: 3/1; week 3: 4/4; week 4: 7/8).

To illustrate scientific interest in the topic, as measured by the number of publications, Figure 2 shows the comparison to the number of COVID-19–related publications during the first 12 weeks of 2020 [11].

### Journals

The papers were published in journals covering a wide range of scientific disciplines. Table 3 shows an overview of the specialty distribution of journals published on ChatGPT. The current impact factor of the represented journals ranged from 0 to 96.216, with a median of 5.144 (IQR 3.352-11.325). Overall, 45.6% (n=73) of all articles were published “traditionally” in contrast to 54.4% (n=87) that were published as open access. Of those, 5% (n=8) of “open access” publications were provided on preprint servers in advance. Data on publication speed were accessible in 33.1% (n=53) of all evaluated articles. The average time to publication was 16 days, ranging from 4 to 83 days.

**Table 3:** Disciplines of journals published on ChatGPT (N=160).

Discipline of first authors	Values, (%)
Clinical medicine	72 (45)
Other	44 (27.5)
Education	19 (11.9)
Theoretical medicine	19 (11.9)
Nature sciences	6 (3.8)

### Authors

The authors of the reviewed papers had a median of 5 (IQR 1-12; range 0-94) first and a median of 1 (IQR 0-6; range 0-61) last authorships in the years spanning 2020 to 2022. Their area of expertise spanned all medical specialties, including science journalism, bioinformatics, nursing, humanities, economics, and law. Table 4 gives an overview of the specialty distribution of the first authors.

**Table 4:** Disciplines of first authors publishing on ChatGPT (N=160).

Journal discipline	Values, n (%)
Clinical medicine	82 (51.3)
Other	33 (20.6)
Theoretical medicine	23 (14.4)
Nature sciences	10 (6.3)
Not determined	7 (4.4)
Education	5 (3.1)

### SWOT Analysis

**Figure 3:**
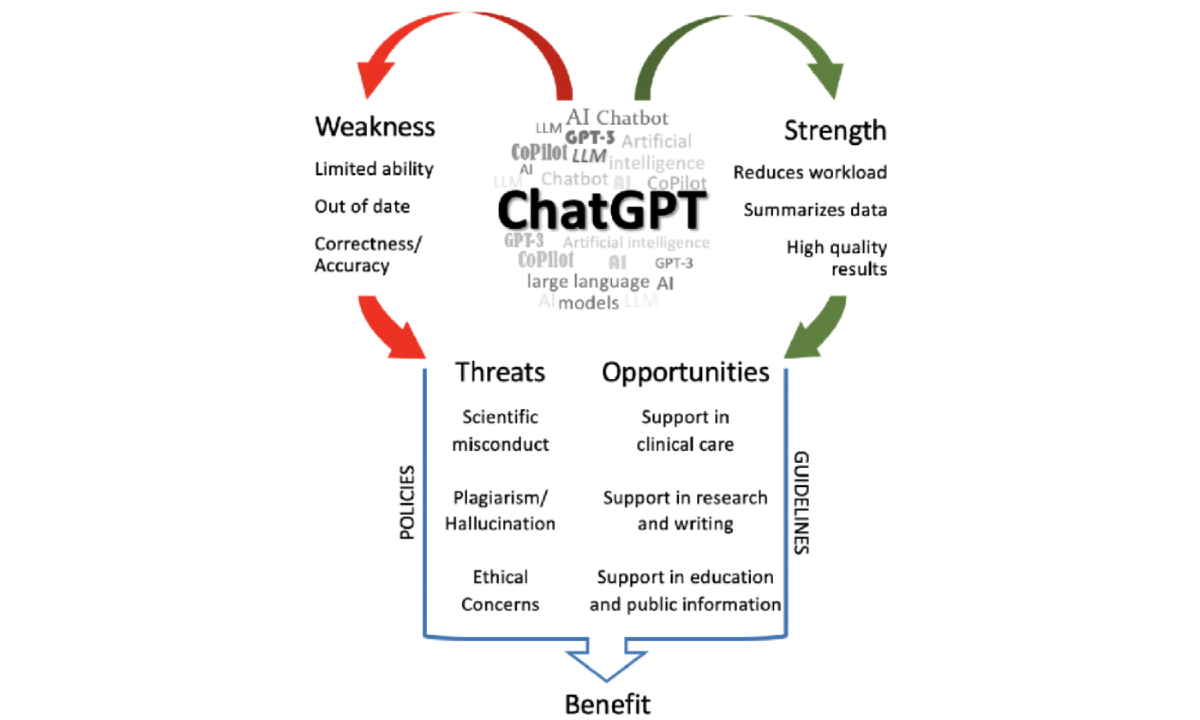
Results, conclusions, and suggestions of SWOT (strengths, weaknesses, opportunities, and threats) analysis of ChatGPT in the medical literature.

We were able to detect 408 quotes (in the 160 papers included) that provided information on strengths, weaknesses, opportunities, and threats. Of those, most were related to weaknesses (n=142, 34.8 %) and least to opportunities (n=68, 16.7%). Quotes on strengths (n=117, 28.7%) and threats (n=81, 19.9%) were mentioned less frequently. Among the most prevalently cited weaknesses were limited abilities [12,13], lack of accuracy/correctness [14,15], citation problems [16,17], and the need for verification [18,19]. Limited abilities in the context of ChatGPT meant not being able to provide reliable facts and sources [12], limitations in understanding complex scientific concepts, or a limited scope of expertise and a lack of accountability [13]. In addition, limited abilities included, for example, the inability to think like a human and evaluate interpersonal aspects, as well as recognize emotions and act on them. Strengths, on the other hand, included reduced workload [14,20-22], data summarization [23], and results rated as positive and high-quality by the authors themselves [15,24-28]. Among the threats captured most frequently were plagiarism (meaning not providing sources), hallucination (meaning making up sources), scientific misconduct, and ethical concerns [25,29-31], whereas major opportunities were seen in supporting different faculties [24,32,33]. For example, Goodman et al [32] mentioned that ChatGPT could improve patient education by personalizing and targeting information to the patient’s education level. Due to the variability in the mentions, we decided to carry out a semiqualitative SWOT analysis. Figure 3 shows the results, conclusions, and suggestions. Raw data on all the aforementioned information can be found in Multimedia Appendix 1.

## Discussion

### Principal Results

To our knowledge, this is the first review of ChatGPT including a SWOT analysis to date, summarizing all articles published in PubMed since ChatGPT launched in November 2022 through the end of March 2023. In addition to a whole series of metric results, the experiences of authors writing about ChatGPT were also critically reviewed in the context of the SWOT analysis. The density and quality of data available at the time of data collection did not allow the use of further, more specific methods and tools to assess the risk of bias. To the best of our knowledge, no similarly comprehensive study on the topic exists to date.

Concerning the article types, it was interesting to see that so far, only 1 RCT has been published about ChatGPT [34]. RCTs on ChatGPT are potentially ambitious and partially difficult to accomplish, but their data are needed to gain more evidence. In this review, the majority of the articles were of a shorter nature (ie, editorials, letters, features, essays, or commentaries).

Journals from the ranks of clinical medicine have published the most articles on ChatGPT, followed by education and others. This resembles the results from authorship. Both aspects (ie, authorship and journal) show the wide application potential for ChatGPT across many specialist areas, as would be expected from a large language model (LLM). When considering the impact factor of the journals, it is interesting to note that some articles were published in journals with no impact factor, although even highly reputable fundamental research journals, such as Science or Nature, and clinical journals, such as the British Medical Journal (BMJ) or the Lancet, have covered the topic. This proves the importance and relevance of ChatGPT in science, education, and clinical work. Out of the 160 papers that were evaluated, 138 (86.3%) were published in journals with an impact factor. Whether a paper was or was not at least partially written with ChatGPT did not have a significant influence on the journal’s impact factor.

Despite the extensive application possibilities of ChatGPT in both medical and nonmedical fields, the publication frequency has not kept pace with the surge seen in COVID-19–related research. This is somewhat surprising considering the significant media attention ChatGPT has received due to remarkable achievements such as passing medical licensing exams, assisting in radiologic decision-making interpretation, and even generating patient clinic letters [4,25,26].

Because ChatGPT is also an event of global significance, we deliberately chose the pandemic as a point of comparison. However, the global health crisis likely served as a stronger catalyst to address the issue, although no relevant difference was seen during the first 4 weeks after the onset of the pandemic compared to the appearance of ChatGPT. Interestingly, it is worth noting that publication speed, if quantifiable, averaged 16 days (ranging from 4 to 83), which is significantly faster than what has been reported in other studies published in biomedical journals [35-37]. However, it is important to recognize that the majority of papers in our review were editorials or commentaries. The quicker publication speed is not as surprising in this context, as these types of publications are easier to produce than larger studies. Furthermore, it is important to note that this statement needs some qualification since corresponding data were only available for about one-third of the publications. This necessitates a more nuanced and less definitive discussion. Alongside the spectrum of journals containing ChatGPT publications, the proportion of preprint and open-access articles should also be considered as influential factors. A multivariate analysis showed that web-based publishing is strongly associated with reduced submission-to-publication time [37]. It must be emphasized that data on submission speed were only available in about one-third of all articles, which is a limiting factor. However, in combination with the higher proportion of quantitative and nonempirical data, we assume that open access and preprints contributed to the fast publication times.

It is difficult to create a comprehensive author profile in this area due to distribution patterns and publication frequencies. However, most authors seem to come from the “clinical medicine” field, which entails working directly with patients. Surprisingly, education, despite being frequently mentioned and an obvious ChatGPT application area, had a lower representation.

Notably, many authors were not newcomers to publishing. Increased public interest in ChatGPT’s medical application seemed to have given them an incentive to conduct research and publish quicker than before. The worth of engaging with ChatGPT is evident from the average impact factor that could be achieved with a publication on this topic, and whether or not an article was written with the help of ChatGPT did not seem to affect the outcome. The median impact of 5.144 (regardless of ChatGPT usage) falls within a range where only 7.9% (n=979) of other journals in a comparison of 13,000 selected scientific journals across 27 major prominent research categories were situated [38].

In addition, despite the allure of using AI in manuscript creation, only slightly under 12% (18/160) of authors used AI, or at least indicated they had. However, since there are no official guidelines for declaring ChatGPT usage in new research, it could be possible that some authors did not disclose its usage. Acknowledging AI use for writing assistance was among the most frequently cited SWOT (which will be discussed later in this section). Thus far, however, ChatGPT usage is not clearly superior or inferior in terms of the impact achieved.

Interestingly, in our SWOT analysis, ChatGPT weaknesses were identified more prominently than strengths, which came in second. It must be said, however, that for the sake of comparability, we weighed the strengths, weaknesses, opportunities, and threats listed by authors equally, regardless of the type of article they were listed in. This is important because essays and opinion pieces were the prevailing types of articles in our analysis, outweighing a more nuanced representation of advantages and disadvantages based on actual trials and scientific research. Given the sheer number of articles that we analyzed, this approach allowed us to make fair comparisons at the time.

When examining the frequency of the SWOT citations, it appeared that many authors provided descriptive accounts of weaknesses and strengths but offered fewer perspectives or ideas for further handling or development of ChatGPT developed from their findings. This is reinforced by the fact that threats were only cited in 19.9% (n=81) papers, and opportunities were mentioned in 16.7% (n=68) compared to weaknesses (n=142, 34.8%).

The SWOT analysis, originally defined as a “strategic planning and strategic management technique used to help a person or organization identify strengths, weaknesses, opportunities, and threats related to business competition or project planning” [39], is used to identify the favorable and unfavorable internal and external factors that impact the objectives of a venture. It is valued for its usability and status as a “tried-and-true tool” for strategic analysis. However, points of criticism include limitations such as potential bias, inconsistency in analysis compliance, and the dominance of certain team members [40-43]. To address some of these shortcomings, we used a modified Delphi process to analyze the quotes. Furthermore, we designed our SWOT analysis as a starting point for discussion, considering it a suitable tool for analyzing ChatGPT in its early stages and generating some ideas for moving forward, particularly in a rapidly changing environment.

Thus far, ChatGPT has been used to write essays, pass exams, translate knowledge for various peer groups, and generate comments on a wide array of topics. However, it has become clear during its application that ChatGPT is, at least until 2021, “apparently “knowledge limited.” This limitation means that it often generates information and facts that are fictional, detectable only by experts with the relevant expertise.

The existing publications on the topic serve a dual purpose. On the one hand, they contribute to the ongoing improvement of AI, making it safer for use in various contexts. On the other hand, they have identified fields where ChatGPT can presumably be applied safely. These applications include summarizing large data sets and producing easily understandable text.

Nonetheless, caution must be exercised when using ChatGPT, as in several cases, sources have been freely invented (hallucination) [14,25] or copied (plagiarism) [16,29,30,44]; therefore, the accurateness of content created by ChatGPT must always be questioned.

Areas unsuitable for ChatGPT’s application include writing scientific papers with references, composing resumes, or writing speeches. In these domains, it has already been shown that ChatGPT can create partially or completely fictitious passages [16,29,30,44], which cannot withstand critical review.

After our concise review, it is clear that ChatGPT serves more as an exploratory tool than a reliable tool for scientific work. This is not inherently negative; playful interactions with ChatGPT can identify strengths and areas for improvement, which developers and programmers can then address. In any case, it is crucial to avoid monopolization (eg, through the displacement of competitors), as it could lead to issues like a lack of transparency in data sources [45] or global commercialization with ethical-economic imbalances [29]. So far, over 30 alternatives for ChatGPT exist, including OpenAI Playground, Jasper Chat, Bard, and Bing AI [46]. Ideally, such large-scale software would be open source.

Another area of major concern is the ability to detect AI-generated scientific output. Existing AI detection software, such as GPTZero, or related products like GLTR, GPTKit, OpenAI, and Output Detector are based on scanning for perplexity (typically lower in AI) and burstiness (typically higher in AI) [47]. Perplexity and burstiness are key AI metrics, aiding our understanding of how LLMs write and how humans can detect AI-generated content. Perplexity evaluates LLM performance, while burstiness is linked to the SD of sentence lengths, making it a measure of text efficiency. Their most obvious, clear limitation is that texts are not analyzed for context but only for writing patterns, potentially allowing AI to go undetected.

Initial data from a comparative study of AI output detectors, plagiarism detectors, and blinded human reviewers show promising results. In that study, AI output detectors successfully identified most AI-generated abstracts, with an area under the receiver operating characteristic (AUROC) curve of 0.94 [48]. However, it is vital to keep pace with the rapid development of new AI interactions like GPT-3 or GPT-4, all while maintaining an alert, skeptical perspective to uphold data integrity.

### The AI Phenomenon Worldwide

ChatGPT, specializing in written conversations, has broad applications across countless domains, underlining the absence of any concrete regulation. As of April 2023, ChatGPT is unavailable in China and other countries with heavy internet censorship, including North Korea, Iran, and Russia. While it is not officially blocked, OpenAI does not allow users from these countries to sign up. Interestingly, several large tech companies in China are developing alternatives [49]. Italy became the first Western country to ban ChatGPT, and various other Western governments, such as Germany, the United Kingdom, and Canada, are currently exploring ways to regulate AI use. The United States has not yet proposed any formal rules to bring oversight to AI technology [49].

### The AI Phenomenon in Medicine

During the review, the threat of being unable to detect the use of ChatGPT in the writing process and the associated lack of reproducibility of its written content were mentioned [33]. For example, Aubignat and Diab [16] cited a study that examined the ability of both a plagiarism detector and a group of reviewers to accurately identify abstracts written by ChatGPT across various journals. Unsurprisingly, they struggled to correctly determine whether the content was original or plagiarist, leading to the conclusion that ChatGPT can write “credible scientific abstracts” [16]. Suggested solutions include consistently mentioning ChatGPT’s possible contribution [24], albeit not as an author [44,50] but more as an “acknowledgement” [51].

Recently, the World Association of Medical Editors (WAME) clearly stated that “AI cannot be an author” and emphasized the responsibility and reproducibility of human authors [52]. A similar stance is also reflected in the criteria of the International Committee of Medical Journal Editors (ICMJE) [53]. Major publishers have started to integrate these recommendations into their policies [54]. Other sources have suggested the inclusion of AI output detectors in the editorial process and clear disclosure if these technologies are used [48].

AI has always fired the human imagination, as can be seen from famous movies like *Star Trek, Star Wars*, *Terminator*, and *Aliens*, always accompanied by a resonating, undefined fear that AI may “overtake” us one day—with potentially deleterious consequences. Despite these easily visualizable and seemingly apocalyptic dangers, one should not dismiss the sheer unlimited and fascinating possibilities offered by AI. Nevertheless, after comparing and evaluating the various strengths, weaknesses, opportunities, and threats posed by ChatGPT, we, the authors, firmly believe that strict and clear regulations on many levels are necessary to fully leverage its potential. In our humble judgment, it seems wise to keep a low profile right now, as ChatGPT itself points out at least some of its weaknesses when asked:

As an artificial intelligence language model, I do not have a role in the discussion about ChatGPT in the medical literature. However, I can provide information and answer questions related to my capabilities and limitations as a language model, as well as share insights on how natural language processing technology is being applied in health care and medical research.

ChatGPT response

## Appendix

Multimedia Appendix 1 PRISMA (Preferred Reporting Items for Systematic Reviews and Meta-Analyses) checklist and raw data from the review.

## Abbreviations

AI: artificial intelligence
AUROC: area under the receiver operating characteristic
BMJ: British Medical Journal
ICMJE: International Committee of Medical Journal Editors
LLM: large language model
MeSH: Medical Subject Headings
ORCID: Open Researcher and Contributor ID
PRISMA: Preferred Reporting Items for Systematic Reviews and Meta-Analyses
RCT: randomized controlled trial
SWOT: strengths, weaknesses, opportunities, and threats
WAIS: Wechsler Adult Intelligence Scale
WAME: World Association of Medical Editors

## Disclaimer

All listed authors declare that all 4 criteria for authorship in the International Committee of Medical Journal Editor (ICMJE) recommendations were met individually. All authors confirm that they had full access to all the data in the study and accept responsibility for submitting the data for publication. All authors claim responsibility and accountability for the originality, accuracy, and integrity of the work. Notably, artificial intelligence (AI) and AI-assisted technologies were used twice as examples and clearly marked as such in the manuscript.

## Data Availability

Without exception, all data collected for this study, including individual participant data and a data dictionary defining each field in the set, will be made available to others. This also includes additional, related documents that will be published and made available upon request by email to the corresponding author.
