# Supplementary material for: A SWOT (Strengths, Weaknesses, Opportunities, and Threats) Analysis of ChatGPT in the Medical Literature: Concise Review
Source: J Med Internet Res. 2023 Nov 16;25:e49368. doi: 10.2196/49368 (PMC10690535; doi:10.2196/49368)
Supplement: Multimedia Appendix 1 [file jmir_v25i1e49368_app1.docx]

Dear Sir or Madam,

attached you will find the two tables for the PRISMA process, which we have completed to the best of our knowledge, our raw data as well as the results of the SWOT analysis.

However, please note that the paper is a concise review and not a systematic review. The data density and quality did not allow for a systematic review at the time the paper was written. The only tools applicable for quality assurance were the high number of reviewers who independently screened and analyzed the data.

For this reason, registration of the work in PROSPERO was also not possible.

Nevertheless, we believe that our work, even as a concise review, meets many of the quality criteria of the PRISMA checklists.

With kind regards

Daniel Gödde on behalf of all authors

1. PRISMA-Checklist

| **Section and Topic** | **Item #** | **Checklist item** | **Location where item is reported** |
| --- | --- | --- | --- |
| **TITLE** | | |  |
| Title | 1 | Identify the report as a systematic review. | Concise review |
| **ABSTRACT** | | |  |
| Abstract | 2 | See the PRISMA 2020 for Abstracts checklist. | See PRISMA Abstract checklist |
| **INTRODUCTION** | | |  |
| Rationale | 3 | Describe the rationale for the review in the context of existing knowledge. | 4 |
| Objectives | 4 | Provide an explicit statement of the objective(s) or question(s) the review addresses. | 5 |
| **METHODS** | | |  |
| Eligibility criteria | 5 | Specify the inclusion and exclusion criteria for the review and how studies were grouped for the syntheses. | 6 |
| Information sources | 6 | Specify all databases, registers, websites, organisations, reference lists and other sources searched or consulted to identify studies. Specify the date when each source was last searched or consulted. | 6 |
| Search strategy | 7 | Present the full search strategies for all databases, registers and websites, including any filters and limits used. | 6 |
| Selection process | 8 | Specify the methods used to decide whether a study met the inclusion criteria of the review, including how many reviewers screened each record and each report retrieved, whether they worked independently, and if applicable, details of automation tools used in the process. | 6 |
| Data collection process | 9 | Specify the methods used to collect data from reports, including how many reviewers collected data from each report, whether they worked independently, any processes for obtaining or confirming data from study investigators, and if applicable, details of automation tools used in the process. | 6/7 |
| Data items | 10a | List and define all outcomes for which data were sought. Specify whether all results that were compatible with each outcome domain in each study were sought (e.g. for all measures, time points, analyses), and if not, the methods used to decide which results to collect. | 6/7 |
|  | 10b | List and define all other variables for which data were sought (e.g. participant and intervention characteristics, funding sources). Describe any assumptions made about any missing or unclear information. | 6/7 |
| Study risk of bias assessment | 11 | Specify the methods used to assess risk of bias in the included studies, including details of the tool(s) used, how many reviewers assessed each study and whether they worked independently, and if applicable, details of automation tools used in the process. | - |
| Effect measures | 12 | Specify for each outcome the effect measure(s) (e.g. risk ratio, mean difference) used in the synthesis or presentation of results. | - |
| Synthesis methods | 13a | Describe the processes used to decide which studies were eligible for each synthesis (e.g. tabulating the study intervention characteristics and comparing against the planned groups for each synthesis (item #5)). | - |
|  | 13b | Describe any methods required to prepare the data for presentation or synthesis, such as handling of missing summary statistics, or data conversions. | - |
|  | 13c | Describe any methods used to tabulate or visually display results of individual studies and syntheses. | - |
|  | 13d | Describe any methods used to synthesize results and provide a rationale for the choice(s). If meta-analysis was performed, describe the model(s), method(s) to identify the presence and extent of statistical heterogeneity, and software package(s) used. | - |
|  | 13e | Describe any methods used to explore possible causes of heterogeneity among study results (e.g. subgroup analysis, meta-regression). | - |
|  | 13f | Describe any sensitivity analyses conducted to assess robustness of the synthesized results. | - |
| Reporting bias assessment | 14 | Describe any methods used to assess risk of bias due to missing results in a synthesis (arising from reporting biases). | - |
| Certainty assessment | 15 | Describe any methods used to assess certainty (or confidence) in the body of evidence for an outcome. | - |
| **RESULTS** | | |  |
| Study selection | 16a | Describe the results of the search and selection process, from the number of records identified in the search to the number of studies included in the review, ideally using a flow diagram. | 9 |
|  | 16b | Cite studies that might appear to meet the inclusion criteria, but which were excluded, and explain why they were excluded. | 9 |
| Study characteristics | 17 | Cite each included study and present its characteristics. | 9 |
| Risk of bias in studies | 18 | Present assessments of risk of bias for each included study. | - |
| Results of individual studies | 19 | For all outcomes, present, for each study: (a) summary statistics for each group (where appropriate) and (b) an effect estimate and its precision (e.g. confidence/credible interval), ideally using structured tables or plots. | - |
| Results of syntheses | 20a | For each synthesis, briefly summarise the characteristics and risk of bias among contributing studies. | 10-14 |
|  | 20b | Present results of all statistical syntheses conducted. If meta-analysis was done, present for each the summary estimate and its precision (e.g. confidence/credible interval) and measures of statistical heterogeneity. If comparing groups, describe the direction of the effect. | 10-14 |
|  | 20c | Present results of all investigations of possible causes of heterogeneity among study results. | 10-14 |
|  | 20d | Present results of all sensitivity analyses conducted to assess the robustness of the synthesized results. | 10-14 |
| Reporting biases | 21 | Present assessments of risk of bias due to missing results (arising from reporting biases) for each synthesis assessed. | 10-14 |
| Certainty of evidence | 22 | Present assessments of certainty (or confidence) in the body of evidence for each outcome assessed. | 10-14 |
| **DISCUSSION** | | |  |
| Discussion | 23a | Provide a general interpretation of the results in the context of other evidence. | 15 |
|  | 23b | Discuss any limitations of the evidence included in the review. | - |
|  | 23c | Discuss any limitations of the review processes used. | - |
|  | 23d | Discuss implications of the results for practice, policy, and future research. | - |
| **OTHER INFORMATION** | | |  |
| Registration and protocol | 24a | Provide registration information for the review, including register name and registration number, or state that the review was not registered. | - |
|  | 24b | Indicate where the review protocol can be accessed, or state that a protocol was not prepared. | A review protocol for this concise review was not prepared. |
|  | 24c | Describe and explain any amendments to information provided at registration or in the protocol. |  |
| Support | 25 | Describe sources of financial or non-financial support for the review, and the role of the funders or sponsors in the review. | 22 |
| Competing interests | 26 | Declare any competing interests of review authors. | 22 |
| Availability of data, code and other materials | 27 | Report which of the following are publicly available and where they can be found: template data collection forms; data extracted from included studies; data used for all analyses; analytic code; any other materials used in the review. | 22 |

*From:*  Page MJ, McKenzie JE, Bossuyt PM, Boutron I, Hoffmann TC, Mulrow CD, et al. The PRISMA 2020 statement: an updated guideline for reporting systematic reviews. BMJ 2021;372:n71. doi: 10.1136/bmj.n71

For more information, visit: <http://www.prisma-statement.org/>

2. PRISMA-Checklist for Abstracts

| **Section and Topic** | **Item #** | **Checklist item** | **Reported (Yes/No)** |
| --- | --- | --- | --- |
| **TITLE** | | |  |
| Title | 1 | Identify the report as a systematic review. | No |
| **BACKGROUND** | | |  |
| Objectives | 2 | Provide an explicit statement of the main objective(s) or question(s) the review addresses. | Yes |
| **METHODS** | | |  |
| Eligibility criteria | 3 | Specify the inclusion and exclusion criteria for the review. | Yes |
| Information sources | 4 | Specify the information sources (e.g. databases, registers) used to identify studies and the date when each was last searched. | Yes |
| Risk of bias | 5 | Specify the methods used to assess risk of bias in the included studies. | No |
| Synthesis of results | 6 | Specify the methods used to present and synthesise results. | Yes |
| **RESULTS** | | |  |
| Included studies | 7 | Give the total number of included studies and participants and summarise relevant characteristics of studies. | Yes |
| Synthesis of results | 8 | Present results for main outcomes, preferably indicating the number of included studies and participants for each. If meta-analysis was done, report the summary estimate and confidence/credible interval. If comparing groups, indicate the direction of the effect (i.e. which group is favoured). | Yes |
| **DISCUSSION** | | |  |
| Limitations of evidence | 9 | Provide a brief summary of the limitations of the evidence included in the review (e.g. study risk of bias, inconsistency and imprecision). | No |
| Interpretation | 10 | Provide a general interpretation of the results and important implications. | Yes |
| **OTHER** | | |  |
| Funding | 11 | Specify the primary source of funding for the review. | Yes |
| Registration | 12 | Provide the register name and registration number. | No |

*From:*  Page MJ, McKenzie JE, Bossuyt PM, Boutron I, Hoffmann TC, Mulrow CD, et al. The PRISMA 2020 statement: an updated guideline for reporting systematic reviews. BMJ 2021;372:n71. doi: 10.1136/bmj.n71

For more information, visit: <http://www.prisma-statement.org/>

3. Raw Data

| **No.** | **Publ. Month/Year** | **Author** | **PMID** | **# Pub as first author** | **# Pub as last author** | **Journal** | **IF** | **Open access (OA) vs. traditional** | **Preprint** | **Article type** | **Qualitative/ quantitative** | **Empiric/ non-empiric** | **Publication speed (in d)** | **Specialty of first author** | **Specialty of first author categorized** | **Specialty of journal** | **Specialty of journal categorized** | **Attempts with ChatGPT** | **Study/Parts written by Chat GPT** |
| --- | --- | --- | --- | --- | --- | --- | --- | --- | --- | --- | --- | --- | --- | --- | --- | --- | --- | --- | --- |
| 1 | Dez 22 | Arif TB | 36809073 | 0 | 0 | Med Educ online | 6 | 1 | 2 | 5 | 0 | 2 | nd | Internal Medicine | 1 | Education | 3 | 1 | 2 |
| 2 | Dez 22 | Stokel-Walker C | 36494443 | 26 | 0 | Nature | 69,504 | 2 | 2 | 6 | 0 | 2 | nd | Editor / Journalist / Pulisher | 5 | All fields of medicine / science | 5 | 2 | 2 |
| 3 | Dez 22 | Castelvecchi D | 36481949 | 94 | 7 | Nature | 69,504 | 2 | 2 | 6 | 0 | 2 | nd | Editor / Journalist / Pulisher | 5 | All fields of medicine / science | 5 | 2 | 2 |
| 4 | Dez 22 | ChatGPT / Zhavoronkov A | 36589923 | 6 | 14 | Oncoscience | x | 1 | 2 | 7 | 0 | 2 | 7 | ChatGPT | 5 | Oncology | 1 | 2 | 1 |
| 5 | Dez 22 | O'Connor S / ChatGPT | 36549229 | 40 | 14 | Nurse Educ Pract | 3,43 | 2 | 2 | 5 | 0 | 2 | nd | Nursing | 1 | Nursing | 1 | 2 | 2 |
| 6 | Jan 23 | Mann DL | 36908674 | 32 | 20 | JACC Basic Transl Sci | 9,531 | 1 | 2 | 7 | 0 | 2 | nd | Internal Medicine | 1 | Translational Medicine / preclinic research | 1 | 2 | 1 |
| 7 | Jan 23 | Duong D | 36789422 | 2 | 0 | medRxiv | x | 1 | 1 | 1 | 2 | 1 | nd | Genetics | 2 | Preprint platform | 5 | 1 | 2 |
| 8 | Jan 23 | Huh S | 36718045 | 16 | 2 | J Educ Eval Health Prof | x | 1 | 2 | 5 | 0 | 2 | 8 | Parasitology | 4 | Education | 3 | 2 | 2 |
| 9 | Jan 23 | Looi MK | 36702491 | 31 | 1 | BMJ | 96,216 | 2 | 2 | 7 | 0 | 2 | nd | Editor / Journalist / Pulisher | 5 | All fields of medicine / science | 5 | 2 | 2 |
| 10 | Jan 23 | Thorp HH | 36701446 | 73 | 0 | Science | 63,714 | 1 | 2 | 5 | 0 | 2 | nd | Editor / Journalist / Pulisher | 5 | All fields of medicine / science | 5 | 1 | 2 |
| 11 | Jan 23 | Shen Y | 36700838 | 2 | 0 | Radiology | 29,146 | 2 | 2 | 5 | 0 | 2 | nd | Data / Computer Science | 5 | Data / Computer Science | 5 | 2 | 2 |
| 12 | Jan 23 | Chatterjee J | 36699746 | 2 | 0 | Patterns | 3,19 | 1 | 2 | 7 | 0 | 2 | nd | Data / Computer Science | 5 | Data / Computer Science | 5 | 1 | 2 |
| 13 | Jan 23 | nal | 36694020 | nal | nal | Nature | 69,504 | 1 | 2 | 5 | 0 | 2 | nd | nal | nal | All fields of medicine / science | 5 | 2 | 2 |
| 14 | Jan 23 | King MR / ChatGPT | 36660590 | 10 | 21 | Cell Mol Bioeng | 3,337 | 1 | 2 | 5 | 0 | 2 | nd | Biomedical Engineering / Informatics / Data Science | 2 | Molecular biology | 4 | 2 | 1 |
| 15 | Jan 23 | Stokel-Walker C | 36653617 | 26 | 0 | Nature | 69,504 | 2 | 2 | 6 | 0 | 2 | nd | Editor / Journalist / Pulisher | 5 | All fields of medicine / science | 5 | 2 | 2 |
| 16 | Jan 23 | Else H | 36635510 | 53 | 2 | Nature | 69,504 | 2 | 2 | 7 | 0 | 2 | nd | Editor / Journalist / Pulisher | 5 | All fields of medicine / science | 5 | 2 | 2 |
| 17 | Jan 23 | Cahan P | 36630899 | 1 | 4 | Stem Cell Reports | 7,294 | 1 | 2 | 5 | 0 | 2 | nd | Biomedical Engineering / Informatics / Data Science | 2 | Stem cell research | 2 | 1 | 2 |
| 18 | Jan 23 | Huh S | 36627845 | 16 | 2 | J Educ Eval Health Prof | x | 1 | 2 | 1 | 2 | 1 | 8 | Parasitology | 4 | Education | 3 | 1 | 2 |
| 19 | Jan 23 | Gordijn B | 36656495 | 7 | 9 | Med Health Care Philos | 1,917 | 1 | 2 | 5 | 0 | 2 | nd | Ethics | 5 | Philosophy | 5 | 1 | 2 |
| 20 | Jan 23 | Ge J | 36972383 | 0 | 0 | Hepatol comm | 5,073 | 2 | 2 | 7 | 4 | 1 | 20 | Internal Medicine | 1 | Internal Medicine | 1 | 1 | 1 |
| 21 | Feb 23 | Tong Y | 36923604 | 6 | 1 | Synth Syst Biotechnol | 4,692 | 2 | 2 | 5 | 0 | 2 | nd | Microbiology | 4 | Biotechnology | 4 | 1 | 1 |
| 22 | Feb 23 | Johnson D | 36909565 | 4 | 12 | Research square | x | 1 | 2 | 1 | 2 | 1 | nd | Oncology | 1 | Preprint platform | 5 | 1 | 2 |
| 23 | Feb 23 | Strunga M | 36900687 | 0 | 0 | Healthcare | 3,16 | 1 | 2 | 2 | 4 | 1 | 57 | Orthopedics / Sports Medicine / Chiropractic | 1 | All fields of medicine / science | 5 | 1 | 2 |
| 24 | Feb 23 | Hosseini M | 36865238 | 0 | 0 | Research square | x | 1 | 1 | 7 | 0 | 2 | nd | Preventive medicine | 1 | Preprint platform | 5 | 1 | 2 |
| 25 | Feb 23 | Rao A | 36865204 | 0 | 0 | medRxiv | x | 1 | 1 | 1 | 1 | 1 | nd | Student | 5 | Preprint platform | 5 | 1 | 2 |
| 26 | Feb 23 | Anderson LB | 36865187 | 2 | 0 | medRxiv | x | 1 | 1 | 1 | 1 | 1 | nd | Environmental medicine | 2 | Preprint platform | 5 | 1 | 2 |
| 27 | Feb 23 | Liu S | 36865144 | 12 | 2 | medRxiv | x | 1 | 1 | 1 | 2 | 1 | nd | Biomedical Engineering / Informatics / Data Science | 2 | Preprint platform | 5 | 1 | 2 |
| 28 | Feb 23 | Homolak J | 36864812 | 13 | 2 | Croat Med J | 2,415 | 1 | 2 | 5 | 0 | 2 | nd | Pharmacology | 2 | Biomedical Engineering / Informatics / Data Science | 2 | 2 | 2 |
| 29 | Feb 23 | Lecler A | 36858933 | 12 | 16 | Diagn Interv Imaging | 7,242 | 2 | 2 | 7 | 0 | 2 | nd | Radiology | 1 | Radiology | 1 | 1 | 2 |
| 30 | Feb 23 | nal | 36854734 | nal | nal | Nature | 69,504 | 2 | 2 | 6 | 0 | 2 | nd | nal | nal | All fields of medicine / science | 5 | 2 | 2 |
| 31 | Feb 23 | Doshi RH | 36853242 | 5 | 0 | Am J Bioeth | 11,229 | 2 | 2 | 5 | 0 | 2 | nd | Student | 5 | Bioethics | 4 | 1 | 2 |
| 32 | Feb 23 | Lee JY | 36842449 | 0 | 0 | J Educ Eval Health Prof | x | 1 | 2 | 2 | 0 | 2 | 43 | Economics / Business / Law | 5 | Education | 3 | 2 | 2 |
| 33 | Feb 23 | Salvagno M | 36841840 | 2 | 0 | BMC Critical Care | 19,334 | 1 | 2 | 5 | 0 | 2 | 26 | Anaesthesiology / Intensive Care Medicine | 1 | Anaesthesiology / Intensive Care Medicine | 1 | 1 | 2 |
| 34 | Feb 23 | Gupta R | 36840479 | 8 | 0 | Aesthet Surg J | 4,485 | 1 | 2 | 5 | 1 | 1 | nd | Surgery | 1 | Surgery | 1 | 1 | 2 |
| 35 | Feb 23 | Hu G | 36840450 | 4 | 5 | Account Res | 3,057 | 2 | 2 | 5 | 0 | 2 | 6 | Editor / Journalist / Pulisher | 5 | Research integrity | 5 | 2 | 2 |
| 36 | Feb 23 | Hirosawa T | 36834073 | 9 | 9 | Int J Environ Res Public Health | 4,614 | 1 | 2 | 1 | 2 | 1 | 21 | General / Family Medicine | 1 | Public Health | 2 | 1 | 2 |
| 37 | Feb 23 | Howard A | 36822213 | 0 | 0 | Lancet Infect Dis | 71,421 | 2 | 2 | 5 | 0 | 2 | nd | Molecular Biology | 4 | All fields of medicine / science | 5 | 2 | 2 |
| 38 | Feb 23 | Brainard J | 36821673 | 28 | 0 | Science | 63,714 | 1 | 2 | 7 | 0 | 2 | nd | Editor / Journalist / Pulisher | 5 | All fields of medicine / science | 5 | 2 | 2 |
| 39 | Feb 23 | Rillig MC | 36821477 | 14 | 35 | Environ Sci Technol | 11,357 | 1 | 2 | 5 | 0 | 2 | 15 | Biology | 4 | Public Health | 2 | 2 | 2 |
| 40 | Feb 23 | Sallam M | 36819954 | 16 | 11 | Cureus | 1,15 | 1 | 2 | 1 | 1 | 1 | 10 | Pathology | 1 | Public Health | 2 | 1 | 2 |
| 41 | Feb 23 | Anderson N | 36816423 | 76 | 42 | BMJ Open Sport Exerc Med | 3,14 | 1 | 2 | 5 | 0 | 2 | nd | Orthopedics / Sports Medicine / Chiropractic | 1 | Orthopedics / Sports Medicine / Chiropractic | 1 | 1 | 2 |
| 42 | Feb 23 | Graf A | 36813155 | 27 | 4 | Neuroscience | 6,709 | 2 | 2 | 7 | 0 | 2 | 13 | Psychiatry / Psychology | 1 | Neurology/Neurosurgery/Neuroradiology/Neurobiology | 1 | 2 | 2 |
| 43 | Feb 23 | Kung TH | 36812645 | 0 | 0 | PLOS Digital Health | x | 1 | 2 | 5 | 2 | 1 | 53 | Anaesthesiology / Intensive Care Medicine | 1 | Education | 3 | 1 | 2 |
| 44 | Feb 23 | Mbakwe AB | 36812618 | 0 | 0 | PLOS Digital Health | x | 1 | 2 | 5 | 0 | 2 | nd | Data / Computer Science | 5 | Education | 3 | 2 | 2 |
| 45 | Feb 23 | Alkaissi H | 36811129 | 7 | 0 | Cureus | 1,15 | 1 | 2 | 5 | 1 | 1 | 13 | Internal Medicine | 1 | General / Family Medicine | 1 | 1 | 2 |
| 46 | Feb 23 | Dahmen J | 36809511 | 18 | 6 | Knee Surg Sports Traumatol Arthrosc | 4,114 | 1 | 2 | 5 | 0 | 2 | 16 | Orthopedics / Sports Medicine / Chiropractic | 1 | Orthopedics / Sports Medicine / Chiropractic | 1 | 1 | 2 |
| 47 | Feb 23 | Huang J | 36799231 | 0 | 0 | J Diabetes Sci Technol | x | 2 | 2 | 5 | 0 | 2 | nd | Neurology/Neurosurgery/Neuroradiology/Neurobiology | 1 | Internal Medicine | 1 | 1 | 2 |
| 48 | Feb 23 | Macdonald C | 36798998 | 0 | 0 | J Glob Health | 7,664 | 1 | 2 | 5 | 0 | 2 | nd | Immunology | 1 | Data / Computer Science | 5 | 1 | 2 |
| 49 | Feb 23 | Rao A | 36798292 | 0 | 0 | medRxiv | x | 1 | 1 | 1 | 1 | 1 | nd | Student | 5 | Preprint platform | 5 | 1 | 2 |
| 50 | Feb 23 | Lubowitz JH | 36797148 | 32 | 11 | Arthroscopy | 4,433 | 2 | 2 | 5 | 0 | 2 | nd | Editor / Journalist / Pulisher | 5 | Surgery | 1 | 2 | 2 |
| 51 | Feb 23 | Gabrielson AT | 36795957 | 12 | 1 | J Urol | 7,641 | 2 | 2 | 5 | 0 | 2 | nd | Urology | 1 | Urology | 1 | 2 | 2 |
| 52 | Feb 23 | Chen TJ | 36791246 | 24 | 7 | J Chin Med Assoc | 3,396 | 1 | 2 | 7 | 0 | 2 | nd | General / Family Medicine | 1 | Biomedical Engineering / Informatics / Data Science | 2 | 2 | 1 |
| 53 | Feb 23 | Tsigaris P | 36786391 | 2 | 2 | Account Res | 3,057 | 2 | 2 | 5 | 0 | 2 | nd | Economics / Business / Law | 5 | Research integrity | 5 | 1 | 2 |
| 54 | Feb 23 | Hallsworth JE | 36786388 | 3 | 4 | Microb Biotechnol | 6,575 | 1 | 2 | 2 | 1 | 1 | 83 | Microbiology | 4 | Microbiology | 4 | 2 | 2 |
| 55 | Feb 23 | Tang G | 36786282 | 3 | 0 | Account Res | 3,057 | 2 | 2 | 5 | 0 | 2 | nd | Editor / Journalist / Pulisher | 5 | Research integrity | 5 | 2 | 2 |
| 56 | Feb 23 | Fijačko N | 36775020 | 11 | 0 | Resuscitation | 6,215 | 2 | 2 | 5 | 0 | 2 | 8 | Anaesthesiology / Intensive Care Medicine | 1 | Anaesthesiology / Intensive Care Medicine | 1 | 1 | 2 |
| 57 | Feb 23 | Ahn C | 36773836 | 5 | 2 | Resuscitation | 6,215 | 2 | 2 | 5 | 0 | 2 | nd | Emergency Medicine | 1 | Anaesthesiology / Intensive Care Medicine | 1 | 1 | 2 |
| 58 | Feb 23 | Krettek C | 36763148 | 4 | 6 | Unfallchirurgie | 0,918 | 1 | 2 | 7 | 0 | 2 | 26 | Surgery | 1 | Surgery | 1 | 1 | 2 |
| 59 | Feb 23 | Lahat A | 36760131 | 0 | 0 | J Telemed Telecare | 6,338 | 2 | 2 | 5 | 0 | 2 | 40 | Internal Medicine | 1 | Telemedicine | 1 | 2 | 2 |
| 60 | Feb 23 | D'Amico RS | 36757199 | 6 | 27 | Neurosurgery | 5,315 | 1 | 2 | 5 | 0 | 2 | nd | Neurology/Neurosurgery/Neuroradiology/Neurobiology | 1 | Neurology/Neurosurgery/Neuroradiology/Neurobiology | 1 | 1 | 2 |
| 61 | Feb 23 | Curtis N / ChatGPT | 36757192 | 1 | 40 | Pediatr Infect Dis J | 3,806 | 2 | 2 | 7 | 0 | 2 | nd | Pediatrics | 1 | Internal Medicine | 1 | 2 | 1 |
| 62 | Feb 23 | Liebrenz M | 36754725 | 5 | 12 | Lancet Digital Health | 36,615 | 1 | 2 | 7 | 0 | 2 | nd | Psychiatry / Psychology | 1 | Digital health | 2 | 2 | 2 |
| 63 | Feb 23 | Patel SB | 36754724 | 0 | 0 | Lancet Digital Health | 36,615 | 1 | 2 | 5 | 0 | 2 | nd | Surgery | 1 | Digital health | 2 | 1 | 2 |
| 64 | Feb 23 | nal | 36754723 | nal | nal | Lancet Digital Health | 36,615 | 1 | 2 | 5 | 0 | 2 | nd | nal | nal | Digital health | 2 | 2 | 2 |
| 65 | Feb 23 | Holzinger A | 36754147 | 5 | 1 | N Biotechnol | 6,49 | 1 | 2 | 5 | 0 | 2 | 25 | Natural Resources and Biosciences | 4 | Biotechnology | 4 | 2 | 2 |
| 66 | Feb 23 | Gilson A | 36753318 | 1 | 0 | JMIR Med Educ | 3,23 | 2 | 2 | 1 | 3 | 1 | 47 | Biomedical Engineering / Informatics / Data Science | 2 | Education | 3 | 1 | 1 |
| 67 | Feb 23 | Moons P | 36752788 | 16 | 23 | Eur J Cardiovasc Nurs | 3,593 | 1 | 2 | 5 | 0 | 2 | nd | Public Health | 2 | Nursing | 1 | 1 | 2 |
| 68 | Feb 23 | Mogali SR | 36749034 | 3 | 6 | Anat Sci Educ | 6,652 | 1 | 2 | 5 | 2 | 1 | 16 | Anatomy / Physiology | 2 | Education | 3 | 1 | 2 |
| 69 | Feb 23 | Yeo-Teh NSL | 36748354 | 6 | 0 | Account Res | 3,057 | 2 | 2 | 5 | 0 | 2 | 11 | Researcher | 5 | Research integrity | 5 | 2 | 2 |
| 70 | Feb 23 | Stokel-Walker C | 36747115 | 26 | 0 | Nature | 69,504 | 2 | 2 | 5 | 0 | 2 | nd | Editor / Journalist / Pulisher | 5 | All fields of medicine / science | 5 | 2 | 2 |
| 71 | Feb 23 | van Dis EAM | 36737653 | 4 | 0 | Nature | 69,504 | 2 | 2 | 7 | 0 | 2 | nd | Psychiatry / Psychology | 1 | All fields of medicine / science | 5 | 2 | 2 |
| 72 | Feb 23 | Kitamura FC | 36728749 | 5 | 3 | RSNA Journal | 29,146 | 2 | 2 | 3 | 0 | 2 | nd | Neurology/Neurosurgery/Neuroradiology/Neurobiology | 1 | Radiology | 1 | 2 | 2 |
| 73 | Feb 23 | Biswas S | 36728748 | 2 | 0 | Radiology | 29,146 | 2 | 2 | 7 | 0 | 2 | nd | Radiology | 1 | Radiology | 1 | 2 | 1 |
| 74 | Feb 23 | Marchandot B | 36915398 | 7 | 0 | Eur Herat J Open | 29,983 | 1 | 2 | 5 | 0 | 2 | 18 | Internal Medicine | 1 | Internal Medicine | 1 | 2 | 1 |
| 75 | Feb 23 | Sng GGR | 36920843 | 0 | 0 | Diabetes Care | 17,242 | 1 | 2 | 5 | 0 | 2 | nd | Internal Medicine | 1 | Internal Medicine | 1 | 1 | 2 |
| 76 | Feb 23 | Hopkins AM | 36808255 | 15 | 21 | JNCI Cancer Spectr | 3,96 | 1 | 2 | 7 | 0 | 2 | 42 | Public Health | 2 | Oncology | 1 | 1 | 2 |
| 77 | Feb 23 | Khan RA | 36950398 | 4 | 7 | Pak J Med Sci | x | 1 | 2 | 7 | 0 | 2 | nd | Pathology | 1 | Education | 3 | 2 | 2 |
| 78 | Feb 23 | Sinha RK | 36968864 | 0 | 0 | Cureus | 1,15 | 1 | 2 | 1 | 3 | 1 | 9 | Pathology | 1 | General / Family Medicine | 1 | 1 | 2 |
| 79 | Feb 23 | Charrois-Durand C | 36999111 | 2 | 0 | Cureus | 1,15 | 1 | 2 | 4 | 0 | 2 | 4 | Oncology | 1 | General / Family Medicine | 1 | 1 | 2 |
| 80 | Mar 23 | Alvero R | 36921837 | 7 | 4 | Fertil Steril | 3,89 | 1 | 2 | 7 | 0 | 2 | nd | Obstetrics and Gynecology | 1 | Obstetrics and Gynecology | 1 | 2 | 1 |
| 81 | Mar 23 | Levin G | 36931435 | 53 | 34 | Am J Obstet Gynecol MFM | 8,679 | 2 | 2 | 1 | 1 | 1 | nd | Obstetrics and Gynecology | 1 | Obstetrics and Gynecology | 1 | 1 | 2 |
| 82 | Mar 23 | Biswas SS | 36920578 | 2 | 0 | Ann Biomed Eng | 4,219 | 2 | 2 | 5 | 0 | 2 | 24 | Radiology | 1 | Biomedical Engineering / Informatics / Data Science | 2 | 2 | 2 |
| 83 | Mar 23 | nal | 36918736 | nal | nal | Nat Med | 87,241 | 2 | 2 | 5 | 0 | 2 | 22 | nal | nal | Translational Medicine / preclinic research | 1 | 2 | 2 |
| 84 | Mar 23 | Lee H | 36916887 | 2 | 0 | Anat Sci Educ | 6,652 | 2 | 2 | 7 | 0 | 2 | nd | Biomedical Engineering / Informatics / Data Science | 2 | Education | 3 | 2 | 2 |
| 85 | Mar 23 | Lahat A | 36914821 | 6 | 6 | Sci Rep | 4,996 | 1 | 2 | 1 | 0 | 2 | 74 | Internal Medicine | 1 | All fields of medicine / science | 5 | 1 | 2 |
| 86 | Mar 23 | Naumova EN | 36914712 | 10 | 21 | J Public Health Policy | 3,53 | 2 | 2 | 5 | 0 | 2 | nd | Nutritional Science | 2 | Public Health | 2 | 2 | 2 |
| 87 | Mar 23 | Potapenko I | 36912780 | 4 | 0 | Acta Opthalm ologica | 3,3 | 2 | 2 | 1 | 0 | 2 | 78 | Ophthalmology | 1 | Ophthalmology | 1 | 1 | 2 |
| 88 | Mar 23 | Zumsteg JM | 36912286 | 0 | 0 | Am J Phys Med Rehabil | 3,412 | 2 | 2 | 4 | 0 | 2 | nd | Orthopedics / Sports Medicine / Chiropractic | 1 | Orthopedics / Sports Medicine / Chiropractic | 1 | 1 | 2 |
| 89 | Mar 23 | Masters K | 36912253 | 9 | 0 | Med Teach | 4,277 | 1 | 2 | 2 | 2 | 1 | nd | Education | 3 | Education | 3 | 2 | 2 |
| 90 | Mar 23 | Beltrami EJ | 36907556 | 10 | 0 | J Am Acad Dermatol | 15,487 | 1 | 2 | 7 | 0 | 2 |  | Dermatology | 1 | Dermatology | 1 | 2 | 2 |
| 91 | Mar 23 | Teixeira da Silva JA | 36906947 | 38 | 15 | Nurse Educ Pract | 3,43 | 2 | 2 | 5 | 0 | 2 | 11 | Researcher | 5 | Education | 3 | 2 | 2 |
| 92 | Mar 23 | Zheng H | 36906169 | 3 | 0 | Am J Med | 5,928 | 1 | 2 | 5 | 0 | 2 | 23 | Internal Medicine | 1 | Internal Medicine | 1 | 1 | 2 |
| 93 | Mar 23 | Goodman RS | 36905924 | 0 | 0 | Med (NY) | x | 2 | 2 | 5 | 0 | 2 | nd | Infection Preventionist and Lab Coordinator | 2 | Translational Medicine / preclinic research | 1 | 2 | 2 |
| 94 | Mar 23 | Morreel S | 36905610 | 9 | 0 | Med Teach | 4,277 | 2 | 2 | 5 | 0 | 2 |  | Health Sciences | 2 | Education | 3 | 2 | 2 |
| 95 | Mar 23 | Najafali D | 36905162 | 3 | 0 | Aesthet Surg J | 4,485 | 1 | 2 | 7 | 0 | 2 |  | Surgery | 1 | Surgery | 1 | 2 | 2 |
| 96 | Mar 23 | Jungwirth D | 36901550 | 2 | 0 | Int J Environ Res Public Health | 4,614 | 1 | 2 | 1 | 0 | 2 | 41 | Public Health | 2 | Public Health | 2 | 1 | 2 |
| 97 | Mar 23 | Siegerink B | 36898252 | 0 | 1 | Nurse Educ Pract | 3,43 | 2 | 2 | 5 | 0 | 2 | nd | Education | 3 | Education | 3 | 2 | 2 |
| 98 | Mar 23 | Ollivier M | 36894785 | 8 | 43 | Knee Surg Sports Traumatol Arthrosc | 4,114 | 2 | 2 | 5 | 0 | 2 | 11 | Orthopedics / Sports Medicine / Chiropractic | 1 | Orthopedics / Sports Medicine / Chiropractic | 1 | 2 | 1 |
| 99 | Mar 23 | Ali SR | 36894409 | 0 | 0 | Lancet Digital Health | 36,616 | 1 | 2 | 7 | 0 | 2 | nd | Surgery | 1 | Digital health | 2 | 1 | 2 |
| 100 | mar 23 | Alberts IL | 36892666 | 0 | 0 | Eur J Nucl Med Mol Imaging | 10,057 | 1 | 2 | 5 | 0 | 2 | 10 | Nuclear medicine | 1 | Nuclear medicine | 1 | 1 | 2 |
| 101 | Mar 23 | Quintans-Júnior LJ | 36888781 | 10 | 19 | Rev Soc Bras Med Trop | 1,581 | 1 | 2 | 5 | 0 | 2 | 14 | Health Sciences | 2 | Tropical medicine | 1 | 2 | 2 |
| 102 | Mar 23 | Checcucci E | 36883768 | 22 | 0 | Minerva Urol Nephrol | 5,214 | 1 | 2 | 7 | 0 | 2 | nd | Surgery | 1 | Urology | 1 | 2 | 2 |
| 103 | Mar 23 | Seghier ML | 36882613 | 2 | 0 | Nature | 69,504 | 2 | 2 | 5 | 0 | 2 | nd | Biomedical Engineering / Informatics / Data Science | 2 | All fields of medicine / science | 5 | 1 | 2 |
| 104 | Mar 23 | Kim SG | 36882591 | 7 | 6 | Maxillofac Plast Reconstr Surg | x | 1 | 2 | 4 | 0 | 2 | nd | Surgery | 1 | Surgery | 1 | 1 | 2 |
| 105 | Mar 23 | Koo M | 36880946 | 5 | 15 | RSNA Journal | 29,146 | 2 | 2 | 5 | 0 | 2 | nd | Nursing | 1 | Radiology | 1 | 2 | 2 |
| 106 | Mar 23 | Ufuk F | 36880943 | 48 | 4 | Radiolog y | 29,146 | 2 | 2 | 5 | 0 | 2 | nd | Radiology | 1 | Radiology | 1 | 2 | 2 |
| 107 | Mar 23 | Scerri A | 36880216 | 5 | 1 | J Clin Nurs | 4,423 | 1 | 2 | 5 | 0 | 2 | nd | Nursing | 1 | Nursing | 1 | 2 | 2 |
| 108 | Mar 23 | Haman M | 36879536 | 2 | 0 | Account Res | 3,057 | 2 | 2 | 5 | 0 | 2 | nd | Humanities | 5 | Research integrity | 5 | 1 | 2 |
| 109 | Mar 23 | Bernstein J | 36877168 | 23 | 4 | Clin Orthop Relat Res | 4,176 | 2 | 2 | 7 | 0 | 2 | nd | Orthopedics / Sports Medicine / Chiropractic | 1 | Surgery | 1 | 1 | 2 |
| 110 | Mar 23 | Nachshon A | 36875254 | 1 | 0 | Cureus | 1,15 | 1 | 2 | 4 | 0 | 2 | 8 | Anaesthesiology / Intensive Care Medicine | 1 | General / Family Medicine | 1 | 2 | 1 |
| 111 | Mar 23 | Šlapeta J | 36872153 | 2 | 27 | Trends Parasitol | 10,523 | 2 | 2 | 7 | 0 | 2 | nd | Veterinary Medicine | 5 | All fields of medicine / science | 5 | 2 | 2 |
| 112 | Mar 23 | Rozencwajg S | 36871626 | 4 | 1 | Anaesth Crit Care Pain Med | 7,025 | 2 | 2 | 5 | 0 | 2 | nd | Anaesthesiology / Intensive Care Medicine | 1 | Scientific writing | 5 | 1 | 1 |
| 113 | Mar 23 | DiGiorgio AM | 36869942 | 8 | 12 | J Med Syst | 4,92 | 1 | 2 | 7 | 0 | 2 | nd | Neurology/Neurosurgery/Neuroradiology/Neurobiology | 1 | Data / Computer Science | 5 | 2 | 2 |
| 114 | Mar 23 | Cascella M | 36869927 | 20 | 39 | J Med Syst | 4,92 | 1 | 2 | 1 | 2 | 1 | 30 | Anaesthesiology / Intensive Care Medicine | 1 | Data / Computer Science | 5 | 1 | 2 |
| 115 | Mar 23 | Boßelmann CM | 36869421 | 4 | 0 | Epilepsia | 6,74 | 1 | 2 | 5 | 0 | 2 | nd | Neurology/Neurosurgery/Neuroradiology/Neurobiology | 1 | Neurology/Neurosurgery/Neuroradiology/Neurobiology | 1 | 2 | 2 |
| 116 | Mar 23 | Hill-Yardin EL | 36868432 | 1 | 10 | Brain Behav Immun | 19,224 | 2 | 2 | 7 | 0 | 2 | 10 | Anatomy / Physiology | 2 | Psychoneurology / Immunology | 2 | 1 | 2 |
| 117 | Mar 23 | Sabry Abdel- Messih M | 36867743 | 0 | 0 | JMIR Med Educ | 3,23 | 1 | 2 | 4 | 0 | 2 | 4 | Toxicology | 2 | Education | 3 | 1 | 2 |
| 118 | Mar 23 | Eysenbach G | 36863937 | 1 | 2 | JMIR Med Educ | 3,23 | 1 | 2 | 5 | 0 | 2 | nd | Editor / Journalist / Pulisher | 5 | Education | 3 | 1 | 2 |
| 119 | Mar 23 | Seney V | 36857593 | 0 | 0 | Nurse Educ | 2,971 | 1 | 2 | 6 | 0 | 2 | nd | Education | 3 | Education | 3 | 2 | 2 |
| 120 | Mar 23 | Biswas SS | 36856927 | 2 | 0 | Ann Biomed Eng | 4,219 | 2 | 2 | 5 | 0 | 2 | 15 | Radiology | 1 | Biomedical Engineering / Informatics / Data Science | 2 | 2 | 2 |
| 121 | Mar 23 | Xue VW | 36856370 | 0 | 0 | Clin Transl Med | 8,554 | 1 | 2 | 7 | 0 | 2 | 8 | Pharmacology | 2 | Translational Medicine / preclinic research | 1 | 2 | 2 |
| 122 | Mar 23 | nd | 36854918 | 0 | 0 | Nature | 69,504 | 2 | 2 | 7 | 0 | 2 | nd | nd | nd | All fields of medicine / science | 5 | 2 | 2 |
| 123 | Mar 23 | Baumgartner C | 36854881 | 6 | 8 | Clin Transl Med | 8,554 | 1 | 2 | 7 | 0 | 2 | nd | Health Sciences | 2 | Translational Medicine / preclinic research | 1 | 2 | 1 |
| 124 | Mar 23 | Owens B | 36807343 | 34 | 1 | Nature | 69,504 | 2 | 2 | 5 | 2 | 1 | nd | Editor / Journalist / Pulisher | 5 | All fields of medicine / science | 5 | 2 | 2 |
| 125 | Mar 23 | Elwood T | 36892853 | 9 | 0 | J Allied Health | 0,646 | 1 | 2 | 5 | 0 | 2 | nd | Editor / Journalist / Pulisher | 5 | Education | 3 | 2 | 2 |
| 126 | Mar 23 | Ferres JML | 36925365 | 2 | 0 | Diagn Interv Imaging | 7,242 | 2 | 2 | 5 | 0 | 2 | nd | Data / Computer Science | 5 | Radiology | 1 | 2 | 1 |
| 127 | Mar 23 | Chavez MR | 36924908 | 0 | 1 | Am J Obstet Gynecol | 9,491 | 2 | 2 | 5 | 0 | 2 | nd | Obstetrics and Gynecology | 1 | Obstetrics and Gynecology | 1 | 2 | 2 |
| 128 | Mar 23 | Grünebaum A | 36924907 | 2 | 0 | Am J Obstet Gynecol | 9,491 | 2 | 2 | 7 | 2 | 1 | 32 | Obstetrics and Gynecology | 1 | Obstetrics and Gynecology | 1 | 1 | 2 |
| 129 | Mar 23 | Castro Nascimento CM | 36926868 | 0 | 0 | J Chem Inf Model | 6,162 | 2 | 2 | 1 | 2 | 1 | nd | Chemistry | 4 | Chemistry | 4 | 1 | 2 |
| 130 | Mar 23 | Johnson SB | 36929393 | 1 | 6 | JNCI Cancer Spectr | 3,96 | 1 | 2 | 1 | 3 | 1 | nd | Oncology | 1 | Oncology | 1 | 1 | 2 |
| 131 | Mar 23 | Choi EPH | 36934624 | 14 | 2 | Nurse Educ Today | 3,906 | 2 | 2 | 7 | 0 | 2 | nd | Nursing | 1 | Nursing | 1 | 2 | 2 |
| 132 | Mar 23 | Kahambing JG | 36934593 | 24 | 1 | Asian J Psychiatr | 13,89 | 2 | 2 | 7 | 0 | 2 | 12 | Psychiatry / Psychology | 1 | Psychiatry / Psychology | 1 | 1 | 2 |
| 133 | Mar 23 | Masters K | 36940462 | 9 | 0 | Med Teach | 4,277 | 2 | 2 | 5 | 0 | 2 | nd | Education | 3 | Education | 3 | 2 | 2 |
| 134 | Mar 23 | Wen J | 36941774 | nd | nd | Clin Transl Med | 8,554 | 2 | 2 | 7 | 0 | 2 | 8 | Economics / Business / Law | 5 | Translational Medicine / preclinic research | 1 | 2 | 2 |
| 135 | Mar 23 | Zhu JJ | 36943179 | 0 | 0 | Environ Sci Technol | 11,357 | 1 | 2 | 5 | 0 | 2 | nd | Civil and Environmental Engineering | 5 | Public Health | 2 | 1 | 2 |
| 136 | Mar 23 | Gupta R | 36943815 | 11 | 0 | Aesthet Surg J | 4,485 | 2 | 2 | 1 | 2 | 1 | nd | Surgery | 1 | Surgery | 1 | 1 | 2 |
| 137 | Mar 23 | nd | 36944743 | 0 | 0 | Nature | 69,504 | 2 | 2 | 5 | 0 | 2 | nd | nd | nd | All fields of medicine / science | 5 | 2 | 2 |
| 138 | Mar 23 | Ali MJ | 36945119 | 76 | 61 | Semin Ophthalmol | 2,246 | 1 | 2 | 5 | 1 | 1 | nd | Ophthalmology | 1 | Ophthalmology | 1 | 1 | 2 |
| 139 | Mar 23 | Shue E | 36945641 | 0 | 0 | bioRxiv | x | 1 | 1 | 4 | 1 | 1 | nd | Microbiology | 4 | Preprint platform | 5 | 1 | 2 |
| 140 | Mar 23 | Yeo YH | 36946005 | 10 | 0 | medRxiv | x | 1 | 2 | 1 | 1 | 1 | nd | Internal Medicine | 1 | Preprint platform | 5 | 1 | 2 |
| 141 | Mar 23 | Sardana D | 36948928 | 4 | 2 | JADA | 3,454 | 2 | 2 | 5 | 0 | 2 | nd | Dentistry | 1 | Dentistry | 1 | 2 | 2 |
| 142 | Mar 23 | Buvat I | 36958855 | 2 | 16 | J Nucl Med | 11,082 | 1 | 2 | 5 | 0 | 2 | nd | Radiology | 1 | Radiology | 1 | 1 | 2 |
| 143 | Mar 23 | Moisset X | 36959062 | 8 | 5 | Rev Neurol | 2,607 | 2 | 2 | 5 | 0 | 2 | 39 | Neurology/Neurosurgery/Neuroradiology/Neurobiology | 1 | Neurology/Neurosurgery/Neuroradiology/Neurobiology | 1 | 2 | 2 |
| 144 | Mar 23 | Aubignat M | 36959064 | 11 | 1 | Rev Neurol | 2,607 | 2 | 2 | 5 | 0 | 2 | nd | Neurology/Neurosurgery/Neuroradiology/Neurobiology | 1 | Neurology/Neurosurgery/Neuroradiology/Neurobiology | 1 | 2 | 2 |
| 145 | Mar 23 | Anders BA | 36960444 | 0 | 0 | Patterns | 3,19 | 1 | 2 | 7 | 0 | 2 | nd | Education | 3 | Data / Computer Science | 5 | 2 | 2 |
| 146 | Mar 23 | Elali FR | 36960451 | 0 | 0 | Patterns | 3,19 | 1 | 2 | 7 | 0 | 2 | nd | Student | 5 | Data / Computer Science | 5 | 1 | 2 |
| 147 | Mar 23 | Janssen BV | 36960954 | 0 | 0 | BJS open | 3,875 | 1 | 2 | 7 | 0 | 2 | 13 | Surgery | 1 | Surgery | 1 | 1 | 1 |
| 148 | Mar 23 | Schorrlepp M | 36961685 | 9 | 1 | MMW Fortschr Med | x | 2 | 2 | 5 | 0 | 2 | nd | General / Family Medicine | 1 | General / Family Medicine | 1 | 1 | 2 |
| 149 | Mar 23 | Odom-Forren J | 36965923 | 11 | 1 | J Perianesth Nurs | 1,352 | 1 | 2 | 7 | 0 | 2 | nd | Nursing | 1 | Nursing | 1 | 1 | 2 |
| 150 | Mar 23 | Benichou L / ChatGPT | 36966950 | 0 | 1 | J Stomato l Oral Maxillofac Surg | 2,48 | 2 | 2 | 7 | 0 | 2 | nd | Surgery | 1 | Surgery | 1 | 2 | 1 |
| 151 | Mar 23 | Subramani M | 36971685 | 7 | 1 | Adv Physiol Educ | 2,39 | 1 | 2 | 5 | 0 | 2 | nd | Anatomy / Physiology | 2 | Anatomy / Physiology | 2 | 2 | 2 |
| 152 | Mar 23 | Ismail A | 36972862 | 23 | 8 | J Am Coll Radiol | 6,24 | 2 | 2 | 7 | 0 | 2 | 31 | Radiology | 1 | Radiology | 1 | 2 | 1 |
| 153 | Mar 23 | Sallam M | 36981544 | 16 | 11 | Healthcare | 3,16 | 1 | 2 | 2 | 4 | 1 | 27 | Pathology | 1 | All fields of medicine / science | 5 | 2 | 2 |
| 154 | Mar 23 | Thomas SP | 36988908 | 32 | 4 | Issues Ment Health Nurs | 1,79 | 1 | 2 | 5 | 0 | 2 | nd | Nursing | 1 | Nursing | 1 | 2 | 2 |
| 155 | Mar 23 | Vaishya R | 36989584 | 14 | 9 | Diabetes Metab Syndr | x | 2 | 2 | 2 | 4 | 1 | 14 | Orthopedics / Sports Medicine / Chiropractic | 1 | Internal Medicine | 1 | 1 | 2 |
| 156 | Mar 23 | Bhatia G | 36989732 | 11 | 1 | Asian J Psychiatr | 13,89 | 2 | 2 | 7 | 0 | 2 | 17 | Psychiatry / Psychology | 1 | Psychiatry / Psychology | 1 | 2 | 2 |
| 157 | Mar 23 | Rahimi F | 36990890 | 19 | 1 | Arch Med Res | 8,323 | 2 | 2 | 7 | 0 | 2 | 36 | Biology | 4 | Biomedical Engineering / Informatics / Data Science | 2 | 2 | 2 |
| 158 | Mar 23 | Borges RM | 36994692 | 7 | 6 | J Biosci | 2,712 | 1 | 2 | 5 | 0 | 2 | nd | Ecology | 5 | Biomedical Engineering / Informatics / Data Science | 2 | 2 | 2 |
| 159 | Mar 23 | Hou W | 36993670 | 5 | 0 | bioRxiv | x | 1 | 1 | 1 | 2 | 1 | nd | Biomedical Engineering / Informatics / Data Science | 2 | Preprint platform | 5 | 1 | 2 |
| 160 | Apr 23 | nal | 36943139 | nal | nal | BJU Int | 5,969 | 2 | 2 | 6 | 0 | 2 | nd | nal | nal | Urology | 1 | 2 | 2 |
|  |  |  |  |  |  |  |  |  |  |  |  |  |  |  |  |  |  |  |  |
|  |  |  |  |  |  |  |  |  |  |  |  |  |  |  |  |  |  |  |  |
|  |  |  |  |  |  |  |  |  |  |  |  |  |  |  |  |  |  |  |  |
|  |  | nal = no author listed |  |  |  |  |  | 1 = OA | 1 = Preprint | 1 = Study | 0 = non-empiric | 1 = empiric |  |  | 1 = Clinical Medicine |  | 1 = Clinical Medicine | 1 = yes | 1 = yes |
|  |  | nd = not determinable |  |  |  |  |  | 2 = Traditional | 2 = No preprint | 2 = Review | 1 = qualitative | 2 = non-empiric |  |  | 2 = Theoretical Medicine |  | 2 = Theoretical Medicine | 2 = no | 2 = no |
|  |  |  |  |  |  |  |  |  |  | 3 = Meta-Analysis | 2 = quantitative |  |  |  | 3 = Education |  | 3 = Education |  |  |
|  |  |  |  |  |  |  |  |  |  | 4 = Case Report | 3 = mixed |  |  |  | 4 = Nature sciences |  | 4 = Nature sciences |  |  |
|  |  |  |  |  |  |  |  |  |  | 5 = Editorial | 4 = review |  |  |  | 5 = Others |  | 5 = Others |  |  |
|  |  |  |  |  |  |  |  |  |  | 6 = News |  |  |  |  |  |  |  |  |  |
|  |  |  |  |  |  |  |  |  |  | 7 = Opinion/Essay |  |  |  |  |  |  |  |  |  |

4. SWOT-Analysis

4.1 Strength

| 1 | - climate scenario generation (SO) |
| --- | --- |
| 2 | - communication and outreach |
| 3 | - Data analysis and interpretation |
| 4 | - decision-making support |
| 5 | - Engineering prompts to obtain high-quality responses |
| 6 | - Few-shot to obtain a fully customized response." |
| 7 | - Sequential Information Retrieval, - Coding, Debugging, and Syntax Explanation |
| 8 | - Writing Improvement, KeyPoints, and Theme Identification. |
| 9 | "16% each are using them to help write research manuscripts, produce presentations or conduct literature reviews" |
| 10 | "AI models may help in clinical decision support, clinical trial recruitment, clinical data management, research support, patient education“ |
| 11 | "answers demonstrate that ChatGPT can be valuable for users who want preliminary information about virtually any topic in the field“ |
| 12 | "Chatbots such as ChatGPT-3 can generate a well- differentiated diagnosis list for common chief complaints." |
| 13 | "ChatGPT automatically creates text based on written prompts from users and is capable to generate various forms of writing, including essays, poems, lyrics for the song, and even an academic essays.“ |
| 14 | "ChatGPT can be a versatile tool for researchers to streamline the writing process” (proper and legal use of ChatGPT is imperative)“ |
| 15 | "ChatGPT can be used in promoting public health“ |
| 16 | "ChatGPT can predict diagnoses and/or recommend specific treatment => raising ethical questions“ (SO) |
| 17 | "ChatGPT can support nursing practice in different ways, nurses need to acknowledge their |
| 18 | "ChatGPT could improve patient education by making it personalized targeted to the patient’s education level“ (SO) |
| 19 | "ChatGPT has numerous potential applications in surgical science, ranging from text generation and improvement to data extraction and clinical decision-making.“ (SO) |
| 20 | "ChatGPT provides accurate information about common cancer myths and misconceptions" |
| 21 | "It is possible to generate clinic letters with a high overall correctness and humanness score with ChatGPT. + reading level that is broadly similar to current real-world human generated letters“ |
| 22 | "Potential application of LLMs: Radiology report generation“ |
| 23 | "Potential impact of LLM on medical education -> benefits: imporved accuracy, increased efficiency, increased student engagement, improved outcomes“ |
| 24 | "Providing information and answering questions, Assessing patient readiness, Providing support and reassurance; educating and training radiologists" |
| 25 | "Researchers who read medical journal abstracts generated by ChatGPT failed to identify one-third of them as written by machine“ (SWOT) |
| 26 | „AI models can automate certain tasks performed by humans, such as data analysis, image acquisition and interpretation. This may increase the efficiency and reduce the workload of health-care professionals, allowing them to focus on higher level tasks that require their expertise and clinical judgement“ |
| 27 | „answers generally eloquent, informed and lacked a significant degree of mistakes |
| 28 | „ChatGPT can be used to write scientific papers about cardiology, chatGPT must be credited" |
| 29 | „ChatGPT can still be a useful tool for checking grammar and syntax errors and refining the language, particularly for non-native speaker." (SO) |
| 30 | „ChatGPT can support surgeon-scientists in various areas, including writing, data collection, and even patient management.“ (SO) |
| 31 | „Language editing issues ok" |
| 32 | „The machine generated text that was clear, comprehensible and could pass for printed literature. Additionally, the machine was kind enough to generate references in the |
| 33 | A chatbot- scientist (SWOT) |
| 34 | Ability to improve scientific language, English grammar, succinctness of text -> reducing language barriers, improving manuscript language quality |
| 35 | Ability to sift through massive amounts of information and produce responses in a conversational and easy-to-understand manner |
| 36 | Able to pass medical licensing exams -shortens discharge times -able to simplify physician’s report |
| 37 | Able to produce philosophical arguments. |
| 38 | Able to solve vignettes accurately, accurate in providing generel medical facts |
| 39 | Abstracts created by ChatGPT were submitted to academic reviewers who only caught 63%of these fakes |
| 40 | Additional use: patient consultations, patient support, marketing |
| 41 | AI can overcome language barriers (for example for chinese foreigners) |
| 42 | AI might be very useful for “big data”" |
| 43 | AI output detector spotted 66% of generated abstracts, human reviewers detected 68% |
| 44 | AI- generated answers displayed high accuracy and completeness scores across various specialties, question types, and difficulty levels. |
| 45 | AI-generated suggestions could be an important complementary part of optimizing CDS alerts, can identify potential improvements to alert logic and support their implementation, and may even be able to assist experts in formulating their own suggestions for CDS improvement (SO) |
| 46 | Assistance and improved access to care“ |
| 47 | Author states that generated article was <1/1,000th the time he needed for article |
| 48 | Benefitting benefience and nonmalficence of patients |
| 49 | BLS & ACLS exams almost succeeded, good tool for self learning and exam preparation |
| 50 | Can be used to delegate tedious jobs |
| 51 | Can reduce health disparities, diminish disease burden, and improve the overall well-being of the population |
| 52 | Cautious about giving recommendations |
| 53 | ChatGPT answered neuroscience questions in English, French, Arabic (polyglot)“ |
| 54 | ChatGPT being used for creating initial drafts, helping in literature research, creating clarity by rewriting sentences, and offering ideas to be used as a tool in an ICU (mentioned 1 time) |
| 55 | ChatGPT can be used as a starting point for students to generate more complex scenarios reflecting the complexity of nursing care and clinical judgement. |
| 56 | ChatGPT can be used as an add-on to constructive writing, reviewing material, and rephrasing the text. |
| 57 | ChatGPT can be used to explain difficult concepts, organise student time and assignments, generate patient scenarios, and practise NCLEX-style questions. |
| 58 | ChatGPT can be utikizised to assist Physician in diagnosis, decision-making and planning of treatment |
| 59 | ChatGPT great tool formatting scientific articles |
| 60 | ChatGPT is better in negative-worded questions |
| 61 | ChatGPT is great writing support tool |
| 62 | ChatGPT passed memorizing exam, need for more relevant education and assessment (SO) |
| 63 | ChatGPT wrote an article on Rapamycin in the context of Pascal's Wager -> demonstrates ChatGPTs potential. |
| 64 | Could be used to create hypotheses and experiments (SO) |
| 65 | Could be useful for personalized medicine approaches, particularly for patients with neurodegenerative conditions (SO) |
| 66 | Could help find new biomarkers |
| 67 | Delivers agreeable arguments („Author also agrees with generated arguments") |
| 68 | Empowers patients to be better informed about their care and facilitates efficient shared decision-making |
| 69 | Free and accessible to the public |
| 70 | Generally gives usefull and correct medical information, finds correct study. |
| 71 | Generate patient-centered education materials for various conditions or procedures. |
| 72 | Good starting point for a “patient education” material but the output requires further revisions and refinements before its being appro-priate for patient use. |
| 73 | Great benefit predicted from large language models in medical diagnostics |
| 74 | Has produced research abstracts good enough that scientists found it hard to spot that a computer had written them (?) |
| 75 | Help with health interventions relying on communication between non- professional peers |
| 76 | Helpful for recruiting patients for clinical trials |
| 77 | Helpful for writing and structure |
| 78 | Helpful in assistance of writing the article |
| 79 | Helpful in clinical decision making e.g. radiology |
| 80 | Helpful in proofreading + adjusting wording |
| 81 | Helpful in time consuming tasks, able to answer specific questions well |
| 82 | Helps draft a framework for each tailored question asked by patients and caregivers, increasing efficiency for healthcare providers |
| 83 | Helps to write case report + extracted information pertinent to the case report when provided with a list of human-picked references |
| 84 | Helps to write case reports, no plagiarism |
| 85 | High accuracy in SATA prompts, accurate in breast cancer detection moreso than mastodynia. |
| 86 | improve research translation |
| 87 | Improving physicians competence |
| 88 | It can answer questions regarding e.g. healthy lifestyle, vaccination, screening and early detection, risk factors reducing and environmental health |
| 89 | LLMs in general - streamline workflow environment, improvement of writing quality, increase representation of non english speaking countries, enhancing environmental literacy |
| 90 | LLMs like ChatGPT can be used to review manuscripts for grammar, spelling, and punctuation errors (to assist human reviewers) (SO) |
| 91 | Makes humans better in exam |
| 92 | Medical education tool to support learning in medical schools |
| 93 | Opportunity to speed up scientific process (innovation, shortened time to publication) (SO) |
| 94 | Patient receive better output when asking ChatGPT than Google/Bing/Yahoo concerning their health (=> ChatGPT does the filtering and recommends a doctors appointment) |
| 95 | Possibilities for the academic progress in nursing education |
| 96 | Possibility to bring open access to high-quality science (writing plain language summaries for not academic settings) |
| 97 | Potential to improve healthcare delivery and enhance patients' lives, particularly through improving communication between patients and healthcare professionals. |
| 98 | Precisely summarizes existing knowledge |
| 99 | Provided various correct responses to diabetes related prompts e.g. in regards to storage of insulin |
| 100 | Provides an easy- to-understand and potentially reliable source of information |
| 101 | Providing instant, accurate, and personalized responses to a wide range of health care questions, not a replacement for human nurses, |
| 102 | Questions on popular topics are easily and correct answered => very specific topics not (SWT) |
| 103 | Software development and data analysis -> improve productivity, workflow for researchers |
| 104 | Streamline the workflow e.g. write letter to insurance provider |
| 105 | Streamline workflow environment -physicans writing have been stripped of emotions and following templated formats, therefore very similar to Chatgpt -can lead to discussion what is considered a “human touch” in writing |
| 106 | Students could make a good argument that AI is not cheating or plagiarism (freely available to everyone, no unfair advantage) |
| 107 | Supports students in learning |
| 108 | There are ongoing updates (SWOT) |
| 109 | Tool to help augment and quicken manuscript writing |
| 110 | Translation |
| 111 | Useable as research tool |
| 112 | Used as digital secretary secretary or for brainstorming |
| 113 | Useful for writing abstracts |
| 114 | Useful in medical education, students should be trained in usage |
| 115 | Uses chatGPT as a tool to support, rather than replace, healthcare professionals in their decision-making process. |
| 116 | Utilizing AI to unburden physicians |
| 117 | Valuable tool in surgical science and clinical care by augmenting, not replacing, human expertise. |

4.2 Weakness

| 1 | ChatGPT better in memorization questions than critical thinking |
| --- | --- |
| 2 | ChatGPT may only serve as starting points for hepatology-specific questions |
| 3 | Urgent need for regulators and health-care professionals to be involved in developing standards for minimum quality and to raise patient awareness of current limitations of emerging AI assistants |
| 4 | Repetitive and lacked depth and insight |
| 5 | ChatGPT cannot provide a whole original blueprint |
| 6 | ChatGPT maths deficiency, makes maths mistakes |
| 7 | An AI program cannot be an author |
| 8 | Answers are inaccurate the more broad the question asked is |
| 9 | Parasitology examination: ChatGPT vs. medical students -> ChatGPT lower performance (60,8% vs. 89,6%) |
| 10 | ChatGPT seems to have currently limited utility in this regard (specialized and trustworthy) |
| 11 | Some responses lack professionalism and insights |
| 12 | Limited in ability to critically discuss results and literature |
| 13 | Does not replace independent literature reviews |
| 14 | ChatGPT has uncertainties and shortcomings dangerous advice was repeatedly given despite prompting |
| 15 | To predict future of diabetes technology major limitations lack of transparency of the data source quality of data used to train the model |
| 16 | Not able to detect situations in which imaging is useless -hallucination: wrong interpretation of provided information -not able to give sources, provides information not asked for |
| 17 | Limited because it only has access to information prior 2021 -cannot replace the human compassion and interpersonal skills that are essential for patient care -current use should be limited to low-risk taska with continued human oversight |
| 18 | Demonstration of AIs to translate (DeepL, ChatGPT) - -DeepL probably leader |
| 19 | ChatGPT needs reliable Internet, Threat: may ot be able to fully understand the context or emotional cues of a conversation and may may not be able to provide care at the level of a human care provider |
| 20 | Does not possess the same capabilities as a human educator |
| 21 | Lacks curiosity, imagination and discovery |
| 22 | Lack of original thought |
| 23 | Lacks nuanced advice with more specific patient information; classifies certain symptoms wrongly |
| 24 | Not able to understand the underlying meaning and context of information |
| 25 | Might not be able to identify the relationsship between different pieces of information in a complex medical situation |
| 26 | Started to write a fictional case report despite having been provided with accurate clinical information earlier, and initially producing a good first-draft of the non- fictional case report requested |
| 27 | Won`t replace anyone |
| 28 | „Limitations that ChatGPT hast regarding promoting public health are e.g. limited accurancy, bias and limitation of data, lack of context, limited engagement and no direct connection to health professionals" |
| 29 | LLM could help to identify research priorities well constructed and original texts only on the surface |
| 30 | „Work that relies solely on ChatGPT’s outputs lacks critical thinking and reasoning skills of a human being, and can potentially be detrimental to the research and impede the science advancement.“ |
| 31 | Unable to "take over" exams for medics; even when learning, ChatGPT has to validate answers |
| 32 | Biased answers, not up to date |
| 33 | Text given by ChatGPT is too shallow to use as a neuroscientist |
| 34 | Review of ChatGPT NCLEX-style questions revealed a focus primarily on prioritisation rather than recognition of assessment cues, identification of problems, implementation of interventions, and evaluation of outcomes. |
| 35 | Limitations in understanding complex scientific concepts - lack of contextual awareness - bias in training data - lack of accountability- limited scope of expertise" |
| 36 | „At present, ChatGPT cannot update the training data in a real-time manner. In addition, it can only give general and vague answers in some existing medical-related conversations diagnoses made by ChatGPT are often not comprehensive and adequate" |
| 37 | Questions on popular topics are easily and correct answered => very specific topics not |
| 38 | Not useful to provide reliable facts and sources |
| 39 | The more complex the programming problem gets the less useful ChatGPT could become |
| 40 | Inability to identify specific cut-off values in the management of cirrhosis and guideline recommendations for surveillance/screenin g of HCC |
| 41 | inability to provide tailored recommendations according to the inquirer's region |
| 42 | May not be robust enough to completely comprehend and handle the complexities of human emotion |
| 43 | Information should be used with caution, validity and accuracy have to be checked |
| 44 | Could lead to automation bias |
| 45 | Algorithmic bias |
| 46 | Cautious and conservative use of ChatGPT advised |
| 47 | Tendency for selection and confirmation bias |
| 48 | Largest barriers to the implementation of ChatGPT in clinical practice are deficits in situational awareness, inference, and consistency |
| 49 | ChatGPTs current form is far from mature to handle scientific writing |
| 50 | "Evidence of its weaknesses observed: A significant drawback is that the data on which the model has been trained are apparently not readily updated. The model assessed seems to not reliably (if at all) source data after 2021 Inability to cite sources or truly understand what the user is asking suggests it has the capability to mislead" |
| 51 | Lack of accountability in decision making (not a decision maker) |
| 52 | Further development is needed to improve there liability and robustness of these tools before clinical integration |
| 53 | Repetitive and lacked depth and insight |
| 54 | Not always up to date, has gaps in knowledge |
| 55 | Unable to access relevant literature and knowledge cut- off up to 2021 |
| 56 | Occasionally inaccurate information will be produced by Chat GPT, knowledge cutoff 2021 |
| 57 | Limited because it only has access to information prior 2021 -cannot replace the human compassion and interpersonal skills that are essential for patient care -current use should be |
| 58 | Biased answers, not up to date |
| 59 | ”Last mile” problem |
| 60 | ChatGPT maths deficiency, makes maths mistakes |
| 61 | Produces credible but incorrect responses (hallucinations) |
| 62 | Answers are inaccurate the more broad the question asked is |
| 63 | ChatGPT exhibiting significant bias when generating Phython code to predict employee seniority based on gender and race |
| 64 | Students and scientists could deceitfully pass off LLM-written text as their own or use LLMs and produce work that is unreliable |
| 65 | Need for being more critical of consumed information |
| 66 | If the experts are not able to determine what’s true, we lose the middleman to guide us through complicated topics” approach to verify accurate information |
| 67 | Fake information endangers patients safety -> journals need more rigorous ... |
| 68 | ChatGPT/LLMs risks of factual inaccuracies, plagiarism, fraud, and copyright infringements |
| 69 | ChatGPT may produce wrong or misleading information |
| 70 | Not able to tell correct dignosis wiht ambiguous scenarios, not able to find the proper dosage for medication, sometimes refuses to give diagnoses even though is hat all information. |
| 71 | Ethical aspects: prejudice and bias in its answers -responses can be more dangerous than bias of search engines (like Google -> users can evaluate sources) |
| 72 | Risk of plagiarism and inaccuracies; imbalance in accessabilits between high- and low income countries if service becomes paying -> regulation in usage of chatbots in scientific writing will soon be required S: application fields in scientific writing: -creating initial draft -helping in literature research -creating clarity by rewriting sentences -offers ideas to be used as a tool in an ICU (creating patient-tailored protocols after acquiring e.g. laboratory values)" |
| 73 | Adverse effects on research, reduced nature experiences, exploitation of the programm and flooding the web with useless information |
| 74 | Concerns with the completeness of the information |
| 75 | Writing errors |
| 76 | Ethical, equity, accuracy and detection concerns |
| 77 | Generated data: mix of true and completely fabricated |
| 78 | Potential of creating false experts |
| 79 | "Creating non existing literature sources, mistakes in creating code, but ChatGPT was able to do a whole study, ethical issues are raised" |
| 80 | ChatGPT/AI challenges factors of publishing like authorship, bias, relevance, accuracy and accountability |
| 81 | Authors tested ChatGPT but it did not perform particularly well -> answers didn’t match with results in the literature, made calculation errors, unable to give literature sources |
| 82 | Risk of bias |
| 83 | Potentially harmful mistakes are made - ChatGPT may give inaccurate information |
| 84 | Does not reliably return accurate information when asked about anatomical facts |
| 85 | Excels in content creation but not always accurate and reliable medical information (SW) |
| 86 | LLM can be misused to generate (racist) hate speech and spam -can´t be trusted for reliable information -concernd about environmental footprint of LLM algorithms |
| 87 | „Potential impact of LLM on medical education + harmful: inaccurate information, job loss in education, ethical questions in the event of a mistake, potential for academic dishonesty" |
| 88 | ChatGPT can’t self- evaluate its output => inaccuracy |
| 89 | „Answers from ChatGPT either clear falsification, incorrect, fabricated tale-like statement,not ready to be used as a trusted source. If everything has to be checked by authors, what's the advantage for ChatGPT?“ |
| 90 | „Although enormous potential, currently remains unreliable due to incorrect or fabricated answers without sourcing.“ |
| 91 | Hallucination: ChatGPT gives clear explanations even to totally wrong answers |
| 92 | "Answers generated to medical questions, some fictitious." |
| 93 | ChatGPT Outpout can contain misinformation and biased language |
| 94 | Parts inaccurate and non-relevant information in responds by ChatGPT |
| 95 | ChatGPT provides parts incorrect output but “solid base to work with” |
| 96 | Erroneous responses => misinformation and serious harm |
| 97 | Sometimes writes plausible sounding but incorrect nonsensical answers |
| 98 | Artificial intelligence hallucination |
| 99 | Hallucinations (GPT models randomly choose an answer when there is a lack of information in the training data) |
| 100 | Potential for the output to be inaccurate or offensive |
| 101 | There has been limited discussion to date regarding the accuracy of the content generated by ChatGPT |
| 102 | „Each response contains information that was either fundamentally wrong or fabricated.“ |
| 103 | "Evidence of its weaknesses observed: A significant drawback is that the data on which the model has been trained are apparently not readily updated. The model assessed seems to not reliably (if at all) source data after 2021 Inability to cite sources or truly understand what the user is asking suggests it has the capability to mislead" |
| 104 | Fabricated information and lack of updated domain knowledge |
| 105 | Recommended to verify sources generated by ChaGPT |
| 106 | Generates sources and references that are not valid but may appear plausible |
| 107 | Author states that references generated by ChatGPT are unfortunately not real |
| 108 | Authors asked ChatGPT to include references in the summary ->returned made-up references, when they asked to include in its summary specific references -> returned the title and authors of completely fabricated publications |
| 109 | "Creating non existing literature sources, mistakes in creating code, but ChatGPT was able to do a whole study, ethical issues are raised" |
| 110 | Not able to detect situations in which imaging is useless -hallucination: wrong interpretation of provided information -not able to give sources, provides information not asked for |
| 111 | Authors tested ChatGPT but it did not perform particularly well -> answers didn’t match with results in the literature, made calculation errors, unable to give literature sources |
| 112 | Cites Pubmed, quotes sound convincing but are actually completely fabricated |
| 113 | Fake references -> AI “hallucinating” or “stochastic parroting” |
| 114 | One has to double-check every evidence (e.g. papers) ChatGPT quotes |
| 115 | „Cannot yet cite sources for documentation and scientific validity" |
| 116 | Chat GPT quotes correct study but gives wrong pub date - doesn’t weigh treatment options but lists them correctly |
| 117 | ChatGPT generated a bibliography with articles not even exsisting |
| 118 | "Evidence of its weaknesses observed: A significant drawback is that the data on which the model has been trained are apparently not readily updated. The model assessed seems to not reliably (if at all) source data after 2021 Inability to cite sources or truly understand what the user is asking suggests it has the capability to mislead" |
| 119 | In 15% different answer to same question |
| 120 | Not necessarily poor performance, it was influenced by several external factors |
| 121 | Unable to access relevant literature and knowledge cut- off up to 2021 |
| 122 | If using the internet that include biased inaccurate and full of outright infos ChatGPT needs to be supervised |
| 123 | Tendency for selection and confirmation bias |
| 124 | Non-english languages may generate better output |
| 125 | "Richness of response and intelligibility of its writing notably inferior to English“„Arabic sometimes inaccurate or nonsensical" |
| 126 | Computationally intensive algorithms (needed to train these programs) require significant amounts of energy -> worries about ecological footprint |
| 127 | Good starting point for a “patient education” material but the output requires further revisions and refinements before its being appro-priate for patient use. |
| 128 | Basic and straightforward questions could be answered adeptly by ChatGPT, but more sophisticated queries will necessitate human-guidance and refinement. |
| 129 | Opportunity cost of relying on ChatGPT (social bias, over reliance) |
| 130 | Medical professionals and patients should be aware of the limitations and actively check AI-generated medical information with trusted sources." |
| 131 | Still require manual checking by a human expert |
| 132 | Limited because it only has access to information prior 2021 -cannot replace the human compassion and interpersonal skills that are essential for patient care -current use should be |
| 133 | Must be used in tandem with human ingenuity and decision-making |
| 134 | ChatGPT ist powerful tool but only with professional (human) supervision |
| 135 | Limited world knowledge after 09/2021 |
| 136 | ChatGPT needs human supervision |
| 137 | Verification needed, texts look authoritative though |
| 138 | Needs to be supervised |
| 139 | Medical professionals and patients should be aware of the limitations and actively check AI-generated medical information with trusted sources." |
| 140 | Evidence of its weaknesses observed: A significant drawback is that the data on which the model has been trained are apparently not readily updated. The model assessed seems to not reliably (if at all) source data after 2021 Inability to cite sources or truly understand what the user is asking suggests it has the capability to mislead" |
| 141 | Still require manual checking by a human expert |
| 142 | Careful with authenticity and verifiability |

4.3 Opportunities

| 1 | Better AI will deliver high-quality care |
| --- | --- |
| 2 | Latest development of GPT models has significantly reduced but hasn’t fully eliminated AI hallucination -New Bing has the best overall performance" |
| 3 | Further development is needed to improve there liability and robustness of these tools before clinical integration. |
| 4 | As LLMs ultimately reflect the contents of its underlying training data, researchers and participants could provide the models with“shared values”by limiting/filtering training data and simultaneously providing active feedback and testing. |
| 5 | „Rigorous evaluation, data diversity, and quality verification by human experts of the training dataset will be required for ChatGPT to become integrated" |
| 6 | Content produced by a conversational AI platform must be validated |
| 7 | The best course of action is to embrace it, use its capabilities to improve our lives, and foster mutually beneficial relationships by evolving it in clinical medicine." |
| 8 | We need an intellectual human mind and a group of policies to cross-check the data generated by such AI systems and control their access. |
| 9 | Recommended to verify sources generated by ChaGPT |
| 10 | Publishers need to acknowledge the tool’s legitimate uses and lay down clear guidelines to avoid |
| 11 | Suggestions for college professors to minimize potential cheating; cheating with AI is unethical; need for being more critical of consumed information |
| 12 | Tools and metrics should be developed to track/tabulate potential harms and misuses to allow for continuous improvement. |
| 13 | While it may be difficult (if not impossible) to mitigate every undesirable behavior of LLMs, with sufficient“guardrails”LLMs could be deployed in a net-beneficial manner to ultimately improve research and practice. |
| 14 | "Need for anti-AI programs to detect plagiarism" |
| 15 | limitations must be considered in the future, as in that it can not replace every aspect of academic research |
| 16 | Novel challenges for the scientific publishing community |
| 17 | "Urgent need for regulators and health-care professionals to be involved in developing standards for minimum quality and to raise patient awareness of current limitations of emerging AI assistants." |
| 18 | ChatGPT in medical practice reveals early promise, but with many considerations for safe and optimal use. |
| 19 | "Currently no guidelines or regulations in place to guide or govern its use.“ |
| 20 | ChatGPT has potential to revolutionize clinical and translational medicine, but we need to develop appropriate strategies to mitigate potential risks and negative outcomes. |
| 21 | "ChatGPT should be used to inform public about CPR in case of emergencies" |
| 22 | Usage for patient information / brochures |
| 23 | Communication and outreach |
| 24 | Good starting point for a “patient education” material but the output requires further revisions and refinements before its being appro-priate for patient use. |
| 25 | ChatGPT may only serve as starting points for hepatology-specific questions. |
| 26 | Basic and straightforward questions could be answered adeptly by ChatGPT, but more sophisticated queries will necessitate human-guidance and refinement. |
| 27 | Potential use in medical education |
| 28 | Potential to supplement human educators |
| 29 | "ChatGPT could improve patient education by making it personalized targeted to the patient’s education level“ |
| 30 | One teaching strategy to encourage clinical judgment in the age of ChatGPT would be to ask students to create a case study based on a ChatGPT-generated NCLEX question about a given concept. Students can then identify assessment cues that lead to problem identification for the given interventions posed by the ChatGPT NCLEX question and develop evaluation strategies to ensure the outcomes were met. Students must provide associated rationales and references to support their clinical judgement as ChatGPT does not typically provide rationale and references in its output. Student feedback using this teaching strategy revealed students were surprised by the ChatGPT functionality. |
| 31 | AI should be integrated into medical school curriculum |
| 32 | ChatGPT passed memorizing exam, need for more relevant education and assessment |
| 33 | "suggests reflecting: questions and assessment; wonders if they should teach students to actively use AI" |
| 34 | Students need to gain AI literacy to be competitive/ effective in the job market |
| 35 | Every person needs to develop their AI literacy |
| 36 | Abstracts created by ChatGPT were submitted to academic reviewers who only caught 63%of these fakes |
| 37 | Author states that generated article was <1/1,000th the time he needed for article |
| 38 | Speed up data analysis, interpretation, and writing |
| 39 | Improve english, applications, etc. |
| 40 | Helpful in writing discharge summaries |
| 41 | „ChatGPT can still be a useful tool for checking grammar and syntax errors and refining the language, particularly for non-native speaker." |
| 42 | ChatGPT as Co-Autor in research |
| 43 | LLMs like ChatGPT can be used to review manuscripts for grammar, spelling, and punctuation errors (to assist human reviewers) |
| 44 | „Potential to revolutionize the way we practice and conduct research in cardiovascular nursing and allied health, generate natural language text, summarize large amounts of data, and answer specific questions" |
| 45 | ChatGPT can aid climate change research by: |
| 46 | Data analysis and interpretation |
| 47 | Climate scenario generation |
| 48 | „AI-based technologies may streamline processes in the research field, but they may also be utilized to undermine legitimate works produced by other authors." |
| 49 | Could be used to create hypotheses and experiments |
| 50 | Opportunity to speed up scientific process (innovation, shortened time to publication) |
| 51 | Discussion with ChatGPT about role of AI in translational medicine |
| 52 | Future LLMs maybe more specialized and trustworthy -> assisting academics in daily work |
| 53 | improving patient outcome and workflow efficiency |
| 54 | Chances: rising accuracy of ChatGPT, potential for human learning in medicine, implementation in medical workflow |
| 55 | ChatGPT will become important virtual assistant to pat. and health-care providers |
| 56 | Could be useful for personalized medicine approaches, particularly for patients with neurodegenerative conditions |
| 57 | "ChatGPT can predict diagnoses and/or recommend specific treatment => raising ethical questions“ |
| 58 | May reduce human errors |
| 59 | "ChatGPT can support nursing practice in different ways, nurses need to acknowledge their limitations and potential risks" |
| 60 | "Potential for AI language models to be used in clinical care" |
| 61 | ChatGPT useful in the future when you have very rare diseases and do not find diagnosis". |
| 62 | Decision-making support |
| 63 | Multi-modal AI (involves combining various data types as images, text, speech, video) has potential to change practice of radiology |
| 64 | "ChatGPT has numerous potential applications in surgical science, ranging from text generation and improvement to data extraction and clinical decision-making.“ |
| 65 | AI-generated suggestions could be an important complementary part of optimizing CDS alerts, can identify potential improvements to alert logic and support their implementation, and may even be able to assist experts in formulating their own suggestions for CDS improvement |
| 66 | "Assistance tool in medical education, research, and clinical management, no replacement for human capability and knowledge" |
| 67 | Supporting clinical practice and scientific writing by establishing a clear understanding of GPT´s abilities and limits |
| 68 | „ChatGPT can support surgeon-scientists in various areas, including writing, data collection, and even patient management.“ |

4.4 Threats

| 1 | Risks of plagiarism, inaccuracies, fraud, and copyright infringements with ChatGPT/LLMs |
| --- | --- |
| 2 | Concerns about the potential use of ChatGPT for plagiarism and academic fraud |
| 3 | ChatGPT's extensive plagiarism |
| 4 | Major concerns are focused on copyright, attribution, plagiarism, and authorship of the articles generated by ChatGPT. |
| 5 | Directly adopting the full text written by ChatGPT may constitute plagiarism and violate the code of conduct for scientific publishing, as originality is the foundation of scientific writing. |
| 6 | ChatGPT flying under the radar of plagiarism checker software |
| 7 | Cannot be screened by plagiarism detection program |
| 8 | Conventional plagiarism detection tools may not be sufficient or sensitive enough to detect plagiarism arising from chatbots |
| 9 | ChatGPT may produce wrong or misleading information |
| 10 | Concerns about the completeness and accuracy of information generated by ChatGPT |
| 11 | ChatGPT making mistakes in math |
| 12 | ChatGPT occasionally producing inaccurate information |
| 13 | Concerns about authenticity and verifiability of ChatGPT-generated content |
| 14 | ChatGPT making writing errors |
| 15 | ChatGPT being unable to make the correct diagnosis in the case of ambiguous clinical scenarios not able to find the proper dosage for medication, sometimes refuses to give diagnoses even though it has all information. |
| 16 | ChatGPT generating a mix of true and completely fabricated data |
| 17 | ChatGPT creating false references and publications |
| 18 | ChatGPT's inability to access relevant literature and knowledge beyond 2021 |
| 19 | Evidence of its weaknesses observed: A significant drawback is that the data on which the model has been trained are apparently not readily updated. The model assessed seems to not reliably (if at all) source data after 2021 Inability to cite sources or truly understand what the user is asking suggests it has the capability to mislead |
| 20 | ChatGPT's limited ability to critically discuss results and literature |
| 21 | ChatGPT's gaps in knowledge |
| 22 | ChatGPT exhibiting significant bias when generating Python code to predict employee seniority based on gender and race |
| 23 | ChatGPTs current form is far from mature to handle scientific writing. |
| 24 | ChatGPT potentially giving dangerous advice |
| 25 | ChatGPT's risks for academic progress in nursing education |
| 26 | Medical professionals and patients should be aware of the limitations and actively check AI-generated medical information with trusted sources. |
| 27 | Largest barriers to the implementation of ChatGPT in clinical practice are deficits in situational awareness, inference, and consistency |
| 28 | Fabricated information and lack of updated domain knowledge. |
| 29 | Lack of accountability in decision making (not a decision-maker) |
| 30 | Good starting point for a “patient education” material but the output requires further revisions and refinements before it's being appropriate for patient use. |
| 31 | Basic and straightforward questions could be answered adeptly by ChatGPT, but more sophisticated queries will necessitate human-guidance and refinement. |
| 32 | Excels in content creation but not always accurate and reliable medical information |
| 33 | ChatGPT needs reliable internet |
| 34 | May not be able to fully understand the context or emotional cues of a conversation and may may not be able to provide care at the level of a human care provider |
| 35 | ChatGPT may only serve as starting points for hepatology-specific questions. |
| 36 | Several critical research needs, especially around inclusion and environmental health justice research |
| 37 | Can´t be trusted for reliable information |
| 38 | Latest development of GPT models has significantly reduced but hasn’t fully eliminated AI hallucination -New Bing has the best overall performance" |
| 39 | There has been limited discussion to date regarding the accuracy of the content generated by ChatGPT |
| 40 | Debate regarding the ethics and acceptability of ChatGPT |
| 41 | Favours high-income countries |
| 42 | Ethical, equity, accuracy, and detection concerns with ChatGPT |
| 43 | Ethical aspects of ChatGPT's potential prejudice and bias in its answers |
| 44 | ChatGPT posing a risk of imbalance in accessibility between high- and low-income countries if the service becomes paying |
| 45 | Computationally intensive algorithms (needed to train these programs) require significant amounts of energy -> worries about ecological footprint |
| 46 | Advanced AI should not be dominated by large tech giants alone |
| 47 | Opportunity cost of relying on ChatGPT (social bias, over-reliance) |
| 48 | Concerns about the potential misuse of ChatGPT and who is responsible for potential damage |
| 49 | ChatGPT potentially being used for political mayhem |
| 50 | ChatGPT potentially making spam, ransomware, and other malicious outputs easier to produce |
| 51 | ChatGPT posing a threat to the intellectual integrity of nursing education by outsourcing writing and thinking |
| 52 | "Currently no guidelines or regulations in place to guide or govern its use.“ |
| 53 | Poor data management is scientific misconduct and should be avoided at all costs |
| 54 | Scientific journals that accept ChatGPT’s involvement in writing will face a significant increase in the number of retractions of published articles and a loss of credibility for the journals. |
| 55 | Students will be outsourcing their writing and their thinking |
| 56 | Publishers need to acknowledge the tool’s legitimate uses and lay down clear guidelines to avoid |
| 57 | Students and scientists could deceitfully pass off LLM-written text as their own or use LLMs and produce work that is unreliable (?) |
| 58 | Abuse |
| 59 | Students possibly cheating withChatGPT |
| 60 | LLM can be misused to generate (racist) hate speech and spam |
| 61 | Google posted a statement that ChatGPT will impose a threat in the near future |
| 62 | Fake information endangers patients safety -> journals need more rigorous |
| 63 | The model assessed seems to not reliably (if at all) source data after 2021 Inability to cite sources or truly understand what the user is asking suggests it has the capability to mislead |
| 64 | ChatGPT's potential to create false experts |
| 65 | ChatGPT's potential to replace physicians |
| 66 | ChatGPT being a chatbot-scientist |
| 67 | Will eventually replace humans |
| 68 | Has produced research abstracts good enough that scientists found it hard to spot that a computer had written them |
| 69 | If the experts are not able to determine what’s true, we lose the middleman to guide us through complicated topics” approach to verify accurate information |
| 70 | Verification needed, texts look authoritative though |
| 71 | Nearly impossible to detect generated text |
| 72 | "Researchers who read medical journal abstracts generated by ChatGPT failed to identify one-third of them as written by machine“ |
| 73 | Abstracts created by ChatGPT were submitted to academic reviewers who only caught a portion of them |
| 74 | Nearly impossible to detect generated text |
| 75 | No software so far can consistently detect the synthetic text |
| 76 | GPT detectors couldn’t recognize that essay was written by AI |
| 77 | Abstracts written by ChatGPT fooled scientists and were published as preprints |
| 78 | AI output detector spotted 66% of generated abstracts, human reviewers detected 68% |
| 79 | Incorrectly identified 32% of generated abstracts as real and 14% of genuine abstracts as generated |
| 80 | Worrisome effects on writing scientific papers |
| 81 | Abstracts created by ChatGPT were submitted to academic reviewers who only caught 63%of these fakes |
